# Explainable Machine Learning Models Using Robust Cancer Biomarkers Identification from Paired Differential Gene Expression

**DOI:** 10.3390/ijms252212419

**Published:** 2024-11-19

**Authors:** Elisa Díaz de la Guardia-Bolívar, Juan Emilio Martínez Manjón, David Pérez-Filgueiras, Igor Zwir, Coral del Val

**Affiliations:** 1 Department of Computer Science and Artificial Intelligence, Andalusian Research Institute in Data Science and Computational Intelligence (DaSCI), University of Granada, 18071 Granada, Spain; 2 Instituto de Investigación Biosanitaria ibs.GRANADA, Complejo Hospitales Universitarios de Granada, Niversidad de Granada, 18012 Granada, Spaindavidperezfil@correo.ugr.es (D.P.-F.); 3 Research Institute in Data Science and Computational Intelligence (DaSCI), University of Granada, 18016 Granada, Spain

**Keywords:** robust biomarkers, machine learning, carcinoma, gene panels

## Abstract

In oncology, there is a critical need for robust biomarkers that can be easily translated into the clinic. We introduce a novel approach using paired differential gene expression analysis for biological feature selection in machine learning models, enhancing robustness and interpretability while accounting for patient variability. This method compares primary tumor tissue with the same patient’s healthy tissue, improving gene selection by eliminating individual-specific artifacts. A focus on carcinoma was selected due to its prevalence and the availability of the data; we aim to identify biomarkers involved in general carcinoma progression, including less-researched types. Our findings identified 27 pivotal genes that can distinguish between healthy and carcinoma tissue, even in unseen carcinoma types. Additionally, the panel could precisely identify the tissue-of-origin in the eight carcinoma types used in the discovery phase. Notably, in a proof of concept, the model accurately identified the primary tissue origin in metastatic samples despite limited sample availability. Functional annotation reveals these genes’ involvement in cancer hallmarks, detecting subtle variations across carcinoma types. We propose paired differential gene expression analysis as a reference method for the discovering of robust biomarkers.

## 1. Introduction

In medicine, there is a critical need for robust biomarkers that can be easily translated into clinic practice to facilitate patient diagnosis and treatment, thereby easing waiting lists. Traditional classification methods have struggled to accurately categorize carcinomas, given the intricate complexity of these malignancies. Histopathological examination often suffers from imprecise histologic criteria and variability in observer expertise, leading to inconsistencies in classification [1,2]. Recent advancements have seen gene panels emerge as powerful tools, analyzing the expression levels of multiple genes simultaneously to provide valuable insights into subtypes, prognosis, and treatment responses. Bioinformatics tools, particularly knowledge-based approaches, have long been employed to prioritize candidates for further analysis [3]. However, the high number of genes and complexity of biological data necessitate additional tools. Machine learning (ML) techniques have recently gained importance for their ability to accurately diagnose and detect diseases by identifying intricate patterns and relationships among gene expression profiles and clinical outcomes [4]. Transparent, robust, and interpretable machine learning models are crucial for ensuring the reliability of clinical applications, providing researchers and clinicians with insights into the underlying biology and rationale behind the models’ decisions [5].

Despite these advances, there remains a need for methods that can enhance the robustness and interpretability of ML models, particularly in the context of human diseases. Building upon our previous work in identifying early prostate cancer biomarkers [6], we propose a novel approach for creating accurate and interpretable classifiers based on the use of paired differential gene expression analysis (DEA) for biological feature selection in machine learning models. This method compares diseased tissue with the same patient’s healthy tissue, improving gene selection by eliminating individual-specific artifacts [7]. Consequently, this enhances the robustness and interpretability of the machine learning models. Furthermore, by using the SHapley Additive exPlanation (SHAP) [8], we gain deeper insights into the model’s inner workings. SHAP enhances model transparency and reliability by assigning importance values to each input feature for each prediction, making it a valuable tool for explaining supervised machine learning models and assessing individual gene impact [9].

Focusing on carcinoma due to its prevalence and the availability of the data, we aim to identify biomarkers involved in general carcinoma progression. This approach identifies commonalities despite the heterogeneity of different carcinomas while addressing the overrepresentation of certain carcinoma types in the literature. For instance, the PAM50 gene panel [10] is widely utilized in breast cancer classification, and OncoType DX Colon [11] is used in colorectal cancer. These gene panels allow oncologists to tailor treatment strategies to individual patients, minimizing overtreatment and maximizing therapeutic efficacy. However, despite their efficacy, the availability of gene panels remains limited, primarily focusing on well-studied carcinomas, such as breast and colorectal cancer [12]. In this study, general carcinoma pivotal genes were derived from eight different carcinomas, each with paired samples from The Cancer Genome Atlas (TCGA). Using ML techniques, we created two types of classifiers with the same set of selected genes: a robust general carcinoma classifier and a multiclass carcinoma tissue-of-origin classifier. Finally, a functional analysis and a SHAP analysis were performed to interpret the role of the selected genes within the context of carcinoma and the ML model.

This study proposes using paired differential gene expression analysis as a feature selection method for implementing ML models with increased robustness and interpretability, accounting for patients’ interpersonal variability. Through the application of this strategy to eight types of carcinomas, we propose a novel, promising gene panel that distinguishes accurately between healthy and carcinoma tissue and precisely identifies the tissue-of-origin.

## 2. Results

This study proposes a new approach to develop accurate and interpretable classifiers based on the identification of robust biomarkers through paired differential gene expression analysis as feature selection method. This approach reduces the effect of individual variations in the selection of the biomarkers, improving robustness and applicability of machine learning models and allowing the identification of tumor samples with high accuracy as well as the tissue-of-origin for the eight carcinomas used.

### 2.1. Paired Differential Gene Expression Analysis

The independent paired differential gene expression analysis (DEA) conducted resulted in eight distinct lists of differentially expressed genes (DEGs). Each list corresponded to a specific carcinoma tissue-of-origin. The analysis revealed varying counts of DEGs across the different types of carcinomas (Table 1 and Table S1 for detailed results).

We identified a total of 5063 differentially expressed genes (DEGs), where the underexpressed genes consistently outnumbered overexpressed ones across all cancer types. Protein-coding genes constituted a substantial portion of the DEGs, accounting for 88–93% in each carcinoma type (Table S2). Additionally, we observed several non-coding genes, including pseudogenes, antisense RNAs, and long intergenic non-coding RNAs (lincRNAs), which were frequently represented (Table S2).

### 2.2. Feature Selection Based on Paired Differential Gene Expression Analysis

In this study, we selected 27 genes that were differentially expressed in at least seven out of eight distinct carcinoma types analyzed. These selected genes, including ANGPTL1, GSTM5, IQGAP3, UHRF1, CCBE1, MYBL2, CHRDL1, PKMYT1, ABCA8, CTHRC1, PKNOX2, UBE2C, DES, RELN, ADH1B, MT1M, CDT1, FAM111B, SFRP1, C7, GHR, LYVE1, IGSF10, MFAP4, RNF150, and HBB, served as essential features for training our classifiers. The expression levels of these genes were consistently altered in the same direction (either overexpressed or underexpressed) across all carcinoma types (Table S3). Among them, only six genes—MMP11, ANGPTL1, GSTM5, IQGAP3, UHRF1, and CCBE1—were shared across all analyzed carcinomas.

### 2.3. Gene Functional Annotation

**Figure 1:**
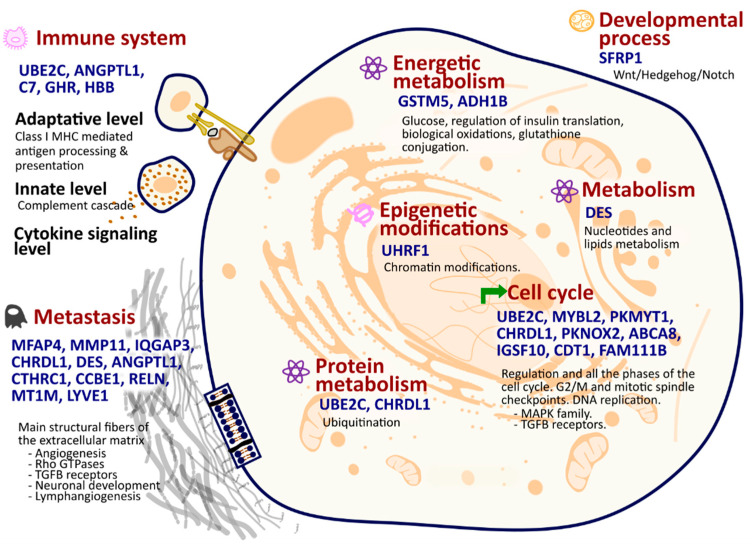
Biological significance: key hallmarks and pathways influenced by the 27 selected genes.

Next, we carried out the functional analysis and annotation of the 27 selected genes. Most of them had been previously associated in the literature with carcinoma, but mostly independently of each other (Table S4). In all cases, their functions encompass the primary alterations or “hallmarks” that normal cells undergo to become cancerous. The range of biological functions include epigenetic modifications, developmental processes, cell cycle regulation, metastasis, immune system interactions, and metabolic pathways (Figure 1, Tables S4 and S5). The UHRF1 gene is involved in chromatin regulation and acetylation, which produces a great impact on tumorigenesis and cancer progression [13]. The SFRP1 gene is key in the developmental process, functioning within the ‘Wnt/Hedgehog/Notch’ signaling pathway, which is essential for cellular development and plays a significant role in tumorigenesis [14]. Genes like UBE2C, MYBL2, PKMYT1, CHRDL1, PKNOX2, ABCA8, IGSF10, CDT1, and FAM111B, as well as the MAPK family and TGFB receptors, are integral to various phases and checkpoints of the cell cycle, orchestrating a complex series of events that ensure proper cell division (Tables S4 and S5). Alterations in the expression of these genes can disrupt the delicate balance of the cell cycle, potentially leading to uncontrolled cell proliferation. Genes such as UBE2C, ANGPTL1, C7, GHR, and HBB are of considerable epigenetic importance and exert a pivotal influence on various facets of immune functionality, including adaptive and innate immunity and cytokine signaling (Tables S4 and S5). Consequently, alterations in their expression patterns could potentially modulate the immune response and thereby contribute to the onset of carcinogenesis. In protein metabolism, ubiquitination pathways are crucial components. Within this context, two genes, UBE2C and CHRDL1, assume pivotal roles in these pathways (Tables S4 and S5). Gene RNF150 seems to also be involved in this context. It is predicted it that enables ubiquitin ligase activity by being involved in the ubiquitin-dependent protein catabolic process [15]. Concerning energetic metabolism, genes such as GSTM5 and ADH1B are important in a range of processes including glucose and insulin translation, biological oxidations, and glutathione conjugation, and the gene DES has a crucial role in the metabolism of nucleotides and lipids.

Finally, numerous genes from the 27 selected are involved in the metastasis hallmark: MFAP4, MMP11, IQGAP3, CHRDL1, DES, ANGPTL1, CTHRC1, CCBE1, RELN, MT1M, and LYVE1. These genes participate in the synthesis and/or degradation pathways of all main structural fibers of the extracellular matrix, including elastic fibers, proteoglycans, and collagen. They also contribute to well-known metastasis-related processes such as angiogenesis, neuronal development, lymph angiogenesis, Rho-GTPases, and TGFB receptor pathways.

### 2.4. The Carcinoma Classifier

To capture intricate patterns and interactions within the data, we selected three different ML methods: Random Forest (RF), Multi-Layer Perceptron (MLP), and XGBoost (Supplementary Methodology Section S1). The specific hyperparameters tested and selected foreach model are listed in supplemental Table S6. In the training set, all models achieved outstanding performance; XGBoost had a weighted average precision, recall, f1-score, and accuracy of 99.50%, MLP had a weighted average precision, f1-score, and accuracy of 96.03%%, and a recall of 96.04%, and RF achieved a perfect score (Table S7, Figure S1).

To assess the feature selection convenience and model’s metrics reproducibility, and assure the inexistence of over-fitting, we repeated the training of the models with three different samplings to create the cross-fold combination using 70% of the sample for training and 30% for test. The results did not change for any of three models. Metric results of XGBoost and RF were similar, and the differences were not significant, although RF gave slightly better results. Given these results, we selected RF as the best model with the following parameters (n_estimators: 100, max_depth: None, min_samples_split: 5, min_sample_leaf: 2, bootstrap: False, and default for all the other parameters). In the test set, the metrics for the RF model were also outstanding, it had an accuracy of 98.07%, weighted average precision of 98.10%, recall of 98.07%, and F1-score of 98.07% (Table S8). In addition to metrics above, we evaluated the Area Under the Receiver Operating Characteristic Curve (AUC-ROC) and the Area Under the Precision-Recall Curve (AUC-PRC) to further verify the model’s performance. The RF model achieved an AUC-ROC of 99.63%, and an AUC-PRC of 99.67%, indicating excellent discrimination ability and precision (Table S8, Figure S1). The excellent metrics obtained on all occasions indicates that the features selected to build the models are robust and relevant, contributing to the strong predictive performance across diverse algorithms.

To assess the carcinoma model’s generalization performance, we used two additional external validation datasets (Table S9). Dataset 1 comprised untrained unpaired samples of the same carcinomas as those used for training the classifier. The RF model, as expected, achieved very high metric values on this dataset, with an accuracy of 99.71%, weighted average recall of 99.71%, and F1-score of 99.85%, respectively. We further validated the model using Dataset 2, consisting of paired samples of diverse carcinoma types distinct from those used in the training data. The RF model demonstrated exceptional accuracy (98.28%), precision (98.25%), recall (98.28%), and F1-score (98.26%). The presence of paired data in Dataset 2 allowed for the calculation of the AUC-ROC (98.99%) and the AUC-PRC (99.92%) (Table S9, Figure S1).

### 2.5. Interpretability of the Carcinoma Classifier

**Figure 2:**
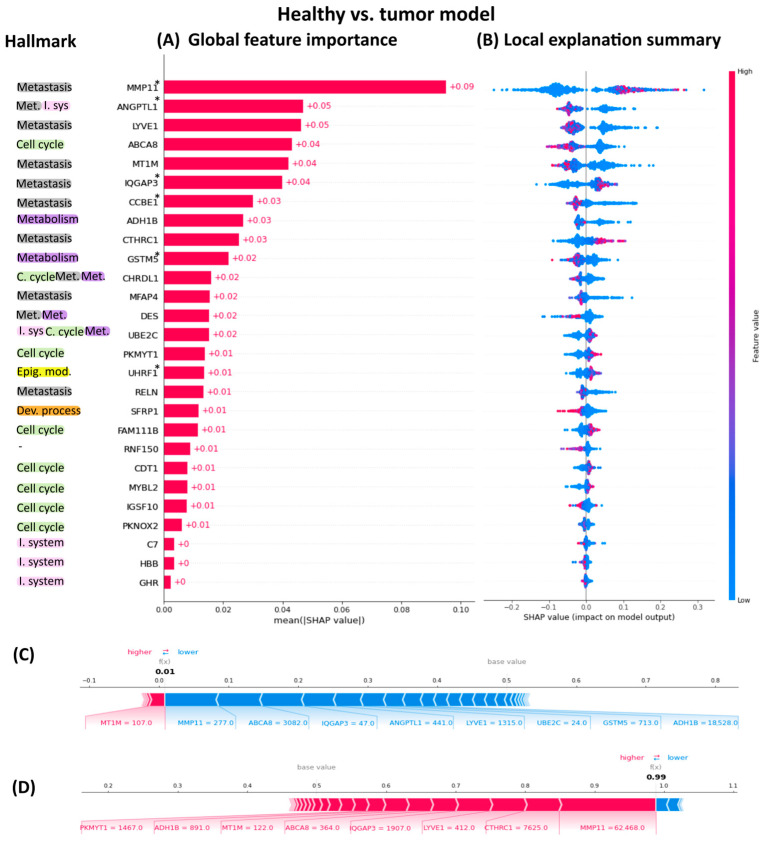
SHAP summary plot for the carcinoma classifier. (**A**) Global feature importance. This plot ranks features by their average SHAP values, highlighting the most influential genes in predicting carcinoma versus healthy tissue. Each bar represents the mean absolute SHAP value of a feature, indicating its overall contribution to the model’s predictions. (**B**) SHAP summary plot. This plot displays SHAP values for each datapoint in the dataset. Each row corresponds to a feature. Colors indicate feature values: red for high and blue for low. Points further to the right have a positive impact in predicting carcinoma on the model’s output, while those to the left have a negative impact. Hallmarks related to each gene are highlighted in the outer left side. * Indicates the six genes common to eight carcinomas (**C**) The SHAP force plot for the healthy sample (ID: TGCA-BH-A1FU-11A-23R-A14D-07) highlights the key genes involved in classifying this sample as healthy. Each gene contributes to the decision in varying proportions, with some genes evaluating the sample across different categories. The final classification is determined by the cumulative contribution of all features. (**D**) The SHAP force plot for the tumor sample (ID: TCGA-BH-A0BC-01A-22R-A084-07) illustrates the significant genes involved in labeling this sample as a tumor. Like the healthy sample, each gene’s contribution varies, and some genes may assess the sample in multiple categories. The overall decision is derived from the aggregate contribution of all features.

The interpretability of our Random Forest carcinoma classifier was assessed using SHAP (SHapley Additive exPlanation) values. To quantify feature importance in the model, we decomposed each prediction into the contributions of individual features using SHAP values. These values provide a unified measure of feature importance, giving insights on how each feature contributes to the model’s predictions. The descending order of the 27 genes’ SHAP values highlights their importance in predicting whether a sample is carcinoma or healthy tissue (Figure 2A).

Figure 2 illustrates the global importance of each gene, with the highest-ranking genes exerting the most significant influence on the model’s predictions. It is noteworthy that the most important genes to distinguish between healthy and tumor samples are related to the metastasis hallmark thought that the data originated from primary tumors. The least important genes were those related to the immune system. The SHAP summary plot reveals the SHAP values associated with all datapoints in our dataset (Figure 2B and Figure S2). Each row corresponds to a specific feature, with color indicating the feature value (red for high values, blue for low values). Individual points represent individual samples. The left/right position of a sample serves as a binary classifier, distinguishing between healthy and tumor samples based solely on that specific feature. This approach facilitates understanding how individual gene values influence the model’s predictions. Additionally, SHAP force plots can illustrate the decision-making process for labeling samples as either healthy or tumor. This decomposition into individual gene contributions is illustrated in Figure 2C,D.

### 2.6. The Carcinoma Tissue-of-Origin Classifier

To validate the robustness of our approach, we employed the same 27 genes selected by the paired differential gene expression analysis (DEA) to construct a multiclass model capable of distinguishing the tissue-of-origin for carcinomas. Specifically, as detailed in Section 2.5, we exclusively utilized tumor samples to train three algorithms: Random Forest (RF), Multilayer Perceptron (MLP), and XGBoost (Supplementary Methodology Section S2). The specific hyperparameters tested and selected foreach model are listed in Supplemental Table S10. Due to class imbalance, we tried 17 different balancing techniques in each algorithm (Table S11). The borderline Synthetic Minority Over-sampling Technique (SMOTE) was determined to be the most effective strategy to equilibrate the class imbalance in XGBoost and MLP, while Adasyn was the best technique for Random Forest algorithm (Table S11). In addition, there were 36 other combinations of algorithm-balancing techniques with an average f1-score of >90%, 18 of them above >95% (Table S11).

Given these results, we selected borderline SMOTE XGBoost as the best multiclass model with the following hyperparameters (learning_rate: 0.3, n_estimators: 100, max_depth: 3, min_child_weight: 1, subsample: 0.7, colsample_bytree = 0.7, gamma = None, and default for all the other parameters). The model’s performance over the train set achieved an average precision, recall, and f1 score of 99.97%, specificity of 99.99%, average Geometric Mean (GEO) of 99.98%, and average Index Balanced Accuracy (IBA) of 99.96% (Table S11). GEO and IBA assess not just the overall accuracy but the fairness and effectiveness of the model across different classes, especially in imbalanced problems.

In the test set, the metrics for the model were also outstanding; we used 452 samples that were left aside from the beginning as the test set. Average precision was 95.04%, average recall was 94.91%, average F1 was 94.92%, average GEO was 96.98%, and average IBA was 93.68% (Table S12). The excellent metrics obtained on all occasions indicate that the features selected to build the models are robust and relevant, contributing to the strong predictive performance across diverse algorithms.

### 2.7. Interpretability and Feature Importance Analysis Using SHAP Values for the Carcinoma Tissue-of-Origin Classifier

To elucidate the differential contributions of 27 key genes within a multiclass machine learning model designed to predict carcinoma tissue-of-origin, we employed SHapley Additive exPlanation (SHAP) to generate summary plots across all model classes (Figures S3–S10). A thorough analysis of these plots reveals distinct gene signatures critical for identifying the specific tumor origins. A notable case is the gene LYVE1, which emerges as the most influential gene in classifying renal carcinoma but ranks as the least significant in gastric carcinoma classification. Thyroid carcinoma exhibited the most divergent gene importance pattern, suggesting a unique gene expression profile distinct from other carcinomas. Although the relative significance of these 27 genes varies across the eight carcinoma types, it is feasible to categorize them into three distinct patterns of influence. For instance, genes such as MYBL2, C7, FAM111B, LYVE, PKMYT, and HBB display comparable importance in classifying both breast and renal carcinomas. Conversely, UHRF1 is consistently the least influential gene in these tissues. A contrasting pattern of similarity emerges among carcinomas originating from uterine, pulmonary, and hepatic tissues, where genes like CTHRC1, ADH1B, GHR, DES, SFRP1, and RNF150 share similar weights. However, the importance of PKMYT1 and HBB differs notably between uterine and both lung and liver origins. Lastly, colorectal and gastric cancers exhibit a shared emphasis on the genes DES, RNF150, and CHRDL1, highlighting a common gene influence pattern.

### 2.8. Metastatic Tissue-of-Origin Prediction Using the Primary Tumor Carcinoma Tissue-Origin Classifier: Proof of Concept

To evaluate the efficacy of the carcinoma tissue-of-origin classifier, initially trained on primary tumor samples, in predicting the tissue origin of metastatic samples, we tested its performance on 16 metastatic samples from TCGA that exhibited expression for the selected 27 genes. The multiclass model demonstrated robust predictive capabilities yielding promising results with a 71% of accuracy for breast cancer, and 87% accuracy for thyroid cancer samples. Despite the heterogeneity of breast cancer and its various subtypes, and the low number of paired data available to learn from some subtypes, the results are notable.

## 3. Materials and Methods

### 3.1. Datasets

#### 3.1.1. Carcinoma Classifier Discovery Dataset

The discovery datasets were obtained from the public database The Cancer Genome Atlas (TCGA; https://portal.gdc.cancer.gov/, accessed on 2 October 2023). We retrieved RNASeq count expression data from multiple carcinoma types: TCGA-COAD, TCGA-BRCA, TCGA-LUAD, TCGA-KIRC, TCGA-STAD, TCGA-LIHC, TCGA-THCA, and TCGA-UCEC. TCGA datasets were retrieved through the TCGAbiolinks package (version 2.14.1) [16].

Datasets covered expression levels of 60,660 genes for 4945 samples across all eight datasets. These datasets had 432 subjects with paired data, a total of 864 samples (Table 2). Paired data comprise samples collected from both the primary tumor and the adjacent healthy tissue within the same individual. Utilizing healthy tissue as an internal control in subsequent analyses minimizes variability attributed to inter-individual differences in baseline gene expression. Only paired data was used for gene selection and for training the general carcinoma classifier.

#### 3.1.2. Carcinoma Tissue-Origin Classifier Discovery Dataset

To construct the carcinoma tissue-of-origin classifier, we used solely tumor samples. The analysis incorporated 4081 unpaired tumor samples and 432 tumor samples from the paired set; the total dataset comprised 4513 samples (Table 2). Of these, 90% were allocated for building the model (*n* = 4061), while the remaining 10% were set aside for validation purposes (*n* = 452) and were never used during the model construction.

#### 3.1.3. Additional Validation Datasets for the Carcinoma Detection Classifier

To assess the model’s generalization performance, we created two additional validation datasets. Dataset 1 contains unpaired tumor samples that were not used during training but belong to the same carcinoma types as those the classifier was trained on (Table 3). Dataset 2 comprises paired samples from carcinoma types not included in the training process, providing RNASeq count expression data for the selected genes: TCGA-READ, TCGA-LUSC, TCGA-KIRP, TCGA-CESC, TCGA-HNSC, TCGA-BLCA, TCGA-PAAD, and TCGA-ESCA. These new eight datasets included 176 healthy and 2556 tumor samples (Table 2).

#### 3.1.4. Carcinoma Tissue-of-Origin Validation Samples

Because of the impossibility to find RNASeq count data for the same carcinoma types and selected genes, carcinoma tissue-type classifier validation was assessed with 452 samples out of the 4513 tumoral samples previously described in the discovery datasets section. These 452 samples had been left aside from the beginning; thus, they were not used for learning the model (Table 4).

### 3.2. Paired Differential Gene Expression Analysis

To avoid interpersonal variability, this means normal differences across people; only paired samples were used for gene selection. Paired differential gene expression analysis (DEA) was performed independently for each cancer dataset comparing healthy versus carcinoma tissue. The analysis was performed using R language version 3.6.2 (https://cran.r-project.org/bin/windows/, accessed on 11 September 2024) and R Studio version 1.2.5019. The eight carcinoma discovery datasets were analyzed using gene filtering with filterByExpr from the edgeR [17] package with default parameters, version 3.28.1. Normalization was done using the trimmed mean of m method (TMM) with the NOISeq package [18], and values were later transformed in the logarithmic scale. Differentially expressed genes (DEGs) between tumor and healthy adjacent samples for each dataset were obtained using a two groups single channel experimental design from the limma package [19], version 3.42.2. Inclusion criteria included a false discovery rate-adjusted *p*-value < 0.01 and at least a 2-fold change.

### 3.3. Feature Selection Based on Paired Differential Gene Expression Analysis

DEGs obtained from the comparison across the eight different cancer datasets indicated in Section 3.2 were sorted by the number of cancers in which they appeared as DEG. Genes that were differentially expressed in at least seven out of the eight analyzed carcinoma paired samples were selected as features to train the machine learning models.

### 3.4. Functional Annotation of Selected Features

Functional annotation and pathway analysis of the genes selected through paired DEA was performed using the GeneCards Suite Pathcards [20] tool (https://www.genecards.org/, accessed on 25 September 2024), the Kyoto Encyclopedia of Genes and Genomes (KEGG) [21], Reactome [22], Gene Ontology [23], WikiPathways [24], and the Human Metabolome Database [25]. Additionally, custom scripts were developed for targeted searches within the PubMed database (https://www.ncbi.nlm.nih.gov/pubmed/, accessed on 25 September 2024) to manually curate the gene-specific literature. Search queries were formulated for each differentially expressed gene using the terms: “[‘cancer’ AND ‘gen symbol’]”, and “[‘carcinoma’ AND ‘gen symbol’]”. Retrieved articles were systematically hand-curated and reviewed. Information was extracted and catalogued in a specialized database.

### 3.5. Machine Learning Development Model

Using the set of genes selected in Section 3.3 we trained two classifiers: one to predict carcinoma presence and the other to identify the tissue-of-origin of the carcinoma. Both classifiers were trained utilizing Python version 3.11 and Spyder version 5.4.3. The methods chosen for our study included the RandomForestClassifier [26], XGBClassifier [27], and MLPClassifier [26]. In all cases, to determine the optimal hyperparameters for each algorithm, we utilized GridSearch [26] in conjunction with a 5-fold cross-validation strategy. Further information on the methods related to machine learning development models are provided in Supplementary Methodology Section S3.

### 3.6. Explainability

In the context of healthcare, models not only need to perform well but also provide insights into the biological and logical foundations of their decisions. One method that has gained prominence is SHAP (SHapley Additive exPlanation) [8]. SHAP values quantify the impact of individual features on a model’s output, enabling both global and local interpretations of feature importance. To enhance model explainability, we generated both global and local summary plots for both classifiers (Supplementary Methodology Section S4).

### 3.7. Evaluation of the Primary Tumor Carcinoma Tissue-Origin Classifier for Predicting Tissue-of-Origin in Metastasic Tissue

In our investigation, we aimed to assess whether the carcinoma tissue-of-origin classifier created from primary tumor samples could accurately predict the tissue-of-origin in metastatic samples. To achieve this, we extracted metastasis-related data from The Cancer Genome Atlas (TCGA) datasets, specifically focusing on samples labeled as “06” for tissue type, which are catalogued as “Metastatic” and refer to samples taken from metastatic destiny sites instead of from primary tumor site. The dataset consisted of a modest 16 samples distributed across eight cancer datasets as follows: TCGA-COAD (*n* = 1), TCGA-BRCA (*n* = 7), and TCGA-THCA (*n* = 8). Despite thorough searches, we were unable to identify any additional open-source cohorts that provide metastasis RNASeq non-transformed count data which also encompass the specific genes used as inputs in our model.

## 4. Discussion

In this study, building upon our previous work in identifying early prostate cancer biomarkers [6], we propose an approach for creating accurate and interpretable classifiers. We applied this methodology to identify a reduced set of common primary tumor biomarkers across eight different carcinomas that could detect, based on gene expression data, the presence of carcinoma or its tissue-of-origin. The rationale behind selecting carcinoma for this proof-of-concept stems from its status as one of the most common prevalent and heterogeneous form of cancers, making it a good example to proof the robustness of the approach. Additionally, its global incidence continues rising, underscoring the need of straightforward diagnostic solutions [28]. This is particularly true for the less-studied carcinomas for which the available data is scarce.

The methodology employed is based in the use of paired differential gene expression analysis (DEA). In this approach, samples of both tumor and healthy tissue are collected from the same patient, allowing for a direct comparison. Genes that are differentially expressed in each type of tumor analyzed are identified, followed by a final selection process. The criterion for this selection is stringent: only genes that are differentially expressed in at least seven of the eight analyzed carcinoma paired samples are chosen as features for training machine learning models. The use of paired DEA helps to overcome patients’ heterogeneity as the healthy tissue serves as subject’s own control [29]. This strategy effectively reduces the noise due to variations in baseline gene expression across different individuals, thereby enhancing the robustness and reproducibility of results by minimizing noise and individual-specific artifacts.

Furthermore, the stringent selection criteria ensure a common small number of features, thereby reducing the search space for machine learning models. High-dimensional data pose challenges due to computational demands, overfitting risks, and interpretability issues [30]. The ‘curse of dimensionality’—having more features than samples—is a common issue in biological data. This challenge is usually overcome by different methods that lead to different genes selection in the same disease [31], often with minimal overlap, hindering consensus and complicating the identification of truly relevant genes for a given disease. We ensure robustness and biological relevance as genes consistently differentially expressed across multiple carcinoma types are more likely to be true positives and associated with key carcinoma mechanisms [32]. By combining these strategies, we aim to minimize potential confounding factors and improve the reliability of our findings by minimizing the risk of overfitting, and facilitating the creation of models that generalizes well to new data [33].

In this study, 27 genes were chosen based on their differential expression in at least seven out of eight distinct carcinoma types analyzed (ANGPTL1, GSTM5, IQGAP3, UHRF1, CCBE1, MYBL2, CHRDL1, PKMYT1, ABCA8, CTHRC1, PKNOX2, UBE2C, DES, RELN, ADH1B, MT1M, CDT1, FAM111B, SFRP1, C7, GHR, LYVE1, IGSF10, MFAP4, RNF150, and HBB). This is the first time that these genes, previously linked to carcinoma in the literature, are presented as a unified panel for general carcinoma characterization. Only MMP11, MYBL2, SFRP1, and UBE2C identified in our study are also featured together as biomarkers in the PAM50 breast cancer 50 gene panel [10]. Additionally, MYBL2 is included in the Colorectal Oncotype DX 12-gene panel [11]. None of the six genes (MMP11, ANGPTL1, GSTM5, IQGAP3, UHRF1, and CCBE1) have previously been categorized as carcinoma biomarkers collectively.

These 27 genes’ functional annotations highlight their significance in the panel, as they represent the primary transformations or “hallmarks” of cancer [34], including epigenetic modifications, developmental processes, cell cycle regulation, metastasis, immune system interactions, and metabolic pathways, as shown in Figure 1. These genes also participate in cancer-related pathways undergoing significant alterations, affecting cell adhesion, ECM receptor interaction, focal adhesion, and PI3K-Akt signaling, among others.

Next, we developed two classifiers—a robust general carcinoma classifier and a multiclass carcinoma tissue-of-origin classifier—using this reduced set of common primary tumor biomarkers as input predictors. In both cases we evaluated the performance of Random Forest, XGBoost, and MLP algorithms, as they can construct interpretable predictive models. Across all metrics, RF and XGBoost slightly outperformed MLP in both cases, achieving in any case very high values. The fact that all different methods achieved good metrics using the selected features suggests that they are robust and relevant, contributing to strong predictive performance across a variety of algorithms.

The general carcinoma classifier outperformed in both test and external validation datasets. Exceptional accuracy was achieved in Dataset 2, which included healthy and cancer samples from carcinoma types not utilized for gene selection. The model demonstrates consistent high predictive power and robustness, even with unseen data of its type, confirming its strong generalization ability.

The multiclass tissue-of-origin classifier for carcinomas, created using the same 27 genes, also demonstrated remarkable precision in differentiating between the eight carcinoma types. This observation is noteworthy because these eight carcinoma subtypes exhibit distinct and complex characteristics based on cancer hallmarks. Despite this complexity our chosen gene panel accurately captures these subtle and sometimes intricate variations in the expression patterns of the 27 genes, enabling precise machine learning-based classification. Although this model currently classifies only the eight carcinoma types used for training, the exceptional results underscore the validity of our methodology and the robustness of the feature selection approach.

By applying AI explainable methods to the carcinoma classifier, we could highlight the importance of expression changes in metastasis hallmark-related genes. These genes serve as indicators of carcinoma progression in primary tissue. There is abundant research that has explored the potential of certain genes to trigger metastatic activity and cancer progression within primary tumors. This continuous process involves several intermediate stages aimed to enhance invasiveness [35,36,37]. As a result, changes in metastasis hallmark genes provide accurate discrimination between healthy and carcinoma tissue. The SHAP analysis of the carcinoma tissue-of-origin classifier identified gene importance patterns that precisely distinguished unique tumor origins. While some genes consistently played significant roles in many carcinomas, others exhibited unique importance. Among all carcinoma, thyroid displayed the most different gene importance pattern and gene expression profile.

We wanted to extend the applicability of the carcinoma tissue-of-origin classifier, so we conducted a proof of concept to determine if the model could accurately classify metastatic tissue to their primary tissue origin. Cancer of unknown primary (CUP) is a metastatic malignancy with an unidentified tissue-of-origin and worse prognosis compared to those with a known origin [38]. Multi-omics approaches have facilitated CUP’s origin identification; however, they are difficult and expensive to transfer to clinic. This proof of concept yielded promising results with a notable accuracy despite the limited sample size to learn from. Unfortunately, due to constraints in sample availability within the TCGA database (sample type code “06”) and the absence of relatable public datasets, increasing the metastatic sample size was not possible. However, it opens an interesting scope into classification of metastatic tissue with a rapid, efficient, and cheap gene panel based on gene expression.

Our proposed strategy has great potential for clinical implications and practical applications. By enhancing robust feature selection and the interpretability of machine learning predictions, we enable clinicians to gain a deeper understanding of the biological mechanisms underlying cancer progression. First, the adoption of paired differential gene expression analysis as a biological feature selection method could facilitate the identification of common causes of carcinoma by removing genes that exhibit high interpersonal variation. For diagnostic purposes, utilizing a set of only 27 pivotal genes to diagnose various carcinomas could optimize the process, making it more efficient and cost-effective. This focused approach has the potential to uncover new drug candidates effective across multiple cancer types, thereby expediting their transition to clinical application. Furthermore, the use of explainable models ensures transparency and facilitates the implementation of precision medicine strategies, ultimately improving patient outcomes through personalized and effective treatments.

The biases in computer science and statistics can be categorized into two primary types of bias that can affect model performance: sample bias and algorithmic bias. Sample bias occurs when the training dataset does not adequately represent the overall population or problem domain, leading to models that may not generalize well beyond the sample data. Algorithmic bias, on the other hand, arises from the design of the algorithm itself, such as the choice of features or optimization functions that might inadvertently favor certain groups or outcomes. 

In this study, we used paired data from only eight carcinomas for training, leaving another eight smaller carcinoma paired datasets for model validation post-process. Additionally, finding RNASeq counts for all 27 genes selected in this study was particularly challenging for external validations and further extension of the tissue-of-origin classifier. To address sample bias, it is crucial to collect more diverse data, use synthetic data augmentation techniques like SMOTE to balance underrepresented groups, and monitor data collection processes to avoid the unintentional exclusion of certain groups. Given the rarity of paired data, we utilized the TCGA, which provides the largest publicly available cohort of paired data in cancer research, encompassing multiple ethnicities. TCGA’s recognition and widespread use as a cancer research resource add credibility and reproducibility to our study. Additionally, we opted for the use of synthetic data augmentation techniques, which addressed at least in part the scarcity of paired data used for feature selection.

To mitigate algorithmic bias, it is possible to implement some key strategies like the use of fairness metrics, such as equal opportunity and demographic parity, to evaluate model performance across different groups, conducting regular audits to detect and correct biased behavior, and implementing transparency and explainability techniques to understand how decisions are made and identify biased patterns. To address and solve algorithmic bias in our study, we implemented several strategies. First, the adoption of paired differential gene expression analysis as a biological feature selection method successfully overcame patient heterogeneity by focusing solely on disease-related changes. This approach improved the fairness of the model by removing the greatest source of variation among different tumor samples. Additionally, we employed cross-validation across subsets to test the model on different population subsets, ensuring its generalizability and fairness. Furthermore, we utilized SHAP (SHapley Additive exPlanation) measures to enhance the explainability of the model. These measures provided insights into how various factors influenced the model’s decisions, allowing us to identify and address any biased patterns. The implementation of these measures to mitigate sample and algorithmic bias proved successful, as evidenced by the development of highly robust models that, despite the limitations of the training samples, were able to generalize effectively to unseen carcinomas, underscoring the algorithm’s capability to handle diverse cancer types, highlighting this strategy as a robust alternative in cancer research.

## 5. Conclusions

In conclusion, we propose the adoption of paired differential gene expression analysis as a biological feature selection method to successfully overcome patients’ heterogeneity and focus only on disease-related changes. We also emphasize the importance of acquiring more paired samples to ensure robust scientific findings, minimize noise, and focus on disease-induced alterations. In addition, considering paired differential gene expression, biological interpretability, and machine learning model results, we propose this novel 27 gene panel as a set of transversal genes that, because of their pivotal role in cancer hallmarks, can accurately distinguish between healthy and carcinoma tissue, and even precisely determine eight carcinoma types. Further studies with suitable datasets from large consortia are needed to confirm the potential of these genes as applicable gene panels in clinic.

## Figures and Tables

**Table 1:** Differential gene expression across the eight TCGA datasets used.

Dataset ID	Overexpressed	Underexpressed	Total
TCGA-COAD	323	760	1083
TCGA-BRCA	263	931	1194
TCGA-LUAD	412	756	1168
TCGA-KIRC	760	833	1593
TCGA-STAD	264	363	627
TCGA-LIHC	163	617	780
TCGA-THCA	318	384	702
TCGA-UCEC	588	995	1583

**Table 2:** Selected samples distribution across cancer types as classified by The Cancer Genome Atlas (TCGA) project. This table provides a breakdown of the total number of samples for each category: Paired Healthy Tissue, Paired Tumor, and Unpaired Tumoral, across the selected carcinoma types.

Dataset ID	Tissue	Paired		Unpaired
		Healthy	Tumor	Tumor
TCGA-COAD	Colorectal adenocarcinoma	41	41	440
TCGA-BRCA	Breast invasive carcinoma	98	98	1013
TCGA-LUAD	Lung adenocarcinoma	57	57	482
TCGA-KIRC	Kidney renal clear cell carcinoma	71	71	470
TCGA-STAD	Gastric adenocarcinoma	33	33	379
TCGA-LIHC	Liver hepatocellular carcinoma	50	50	321
TCGA-THCA	Thyroid carcinoma	59	59	446
TCGA-UCEC	Uterine corpus endometrial carcinoma	23	23	530
		864 (432 healthy/432 tumor)		4081

**Table 3:** Carcinoma external samples as classified by The Cancer Genome Atlas (TCGA) project. Summarizes the total number of samples across eight cancer types for each category, non-paired tumor samples, and healthy and tumor samples.

Dataset ID	Tissue	Carcinoma Classifier Validation		
		Dataset 1	Dataset 2	
		Tumor	Healthy	Tumor
TCGA-COAD	Colorectal adenocarcinoma	440	-	-
TCGA-BRCA	Breast invasive carcinoma	1013	-	-
TCGA-LUAD	Lung adenocarcinoma	482	-	-
TCGA-KIRC	Kidney renal clear cell carcinoma	470	-	-
TCGA-STAD	Gastric adenocarcinoma	379	-	-
TCGA-LIHC	Liver hepatocellular carcinoma	321	-	-
TCGA-THCA	Thyroid carcinoma	446	-	-
TCGA-UCEC	Uterine corpus endometrial carcinoma	530	-	-
TCGA-READ	Rectum Adenocarcinoma	-	10	166
TCGA-LUSC	Lung Squamous Cell Carcinoma	-	51	502
TCGA-KIRP	Kidney Renal Papillary Cell Carcinoma	-	32	290
TCGA-CESC	Cervical Squamous Cell Carcinoma and Endocervical Adenocarcinoma	-	3	304
TCGA-HNSC	Head and Neck Squamous Cell Carcinoma	-	44	520
TCGA-BLCA	Bladder Urothelial Carcinoma	-	19	412
TCGA-PAAD	Pancreatic Adenocarcinoma	-	4	178
TCGA-ESCA	Esophageal Carcinoma	-	13	184
		4081	176	2556

**Table 4:** Carcinoma external samples from various cancer types, as classified by The Cancer Genome Atlas (TCGA) project. Summarizes the 452 samples left aside across all cancer types.

Dataset ID	Tissue	All Tumor
TCGA-COAD	Colorectal adenocarcinoma	48
TCGA-BRCA	Breast invasive carcinoma	111
TCGA-LUAD	Lung adenocarcinoma	54
TCGA-KIRC	Kidney renal clear cell carcinoma	54
TCGA-STAD	Gastric adenocarcinoma	41
TCGA-LIHC	Liver hepatocellular carcinoma	37
TCGA-THCA	Thyroid carcinoma	51
TCGA-UCEC	Uterine corpus endometrial carcinoma	56
		452

## Data Availability

Only publicly available data were used in our study, and data sources and handling of these data are described in the Materials and Methods. Further information is available from the corresponding author upon request.

## Supplementary Materials

The following supporting information can be downloaded at: https://www.mdpi.com/article/10.3390/ijms252212419/s1.

## References

[1] Suster S., Moran C.A. (2005). Problem Areas and Inconsistencies in the WHO Classification of Thymoma. Semin. Diagn. Pathol..

[2] Driessen A., Geboes K.P., Dewit O., Jouret-Mourin A. (2018). Dysplasia in Inflammatory Bowel Disease. Colitis: A Practical Approach to Colon and Ileum Biopsy Interpretation.

[3] Hassani-Pak K., Rawlings C. (2017). Knowledge Discovery in Biological Databases for Revealing Candidate Genes Linked to Complex Phenotypes. J. Integr. Bioinform..

[4] Cuocolo R., Caruso M., Perillo T., Ugga L., Petretta M. (2020). Machine Learning in Oncology: A Clinical Appraisal. Cancer Lett..

[5] Sidak D., Schwarzerová J., Weckwerth W., Waldherr S. (2022). Interpretable Machine Learning Methods for Predictions in Systems Biology from Omics Data. Front. Mol. Biosci..

[6] Díaz de la Guardia-Bolívar E., Barrios-Rodríguez R., Zwir I., Jiménez-Moleón J.J., del Val C. (2022). Identification of Novel Prostate Cancer Genes in Patients Stratified by Gleason Classification: Role of Antitumoral Genes. Int. J. Cancer.

[7] Wang H., Sun Q., Zhao W., Qi L., Gu Y., Li P., Zhang M., Li Y., Liu S.L., Guo Z. (2015). Individual-Level Analysis of Differential Expression of Genes and Pathways for Personalized Medicine. Bioinformatics.

[8] Lundberg S.M., Lee S.I. (2017). A Unified Approach to Interpreting Model Predictions. Adv. Neural Inf. Process. Syst..

[9] Yap M., Johnston R.L., Foley H., MacDonald S., Kondrashova O., Tran K.A., Nones K., Koufariotis L.T., Bean C., Pearson J.V. (2021). Verifying Explainability of a Deep Learning Tissue Classifier Trained on RNA-Seq Data. Sci. Rep..

[10] Perou C.M., Sorlie T., Eisen M.B., Van De Rijn M., Jeffrey S.S., Rees C.A., Pollack J.R., Ross D.T., Johnsen H., Akslen L.A. (2000). Molecular Portraits of Human Breast Tumours. Nature.

[11] Clark-Langone K.M., Sangli C., Krishnakumar J., Watson D. (2010). Translating Tumor Biology into Personalized Treatment Planning: Analytical Performance Characteristics of the Oncotype DX®Colon Cancer Assay. BMC Cancer.

[12] Qian Y., Daza J., Itzel T., Betge J., Zhan T., Marmé F., Teufel A. (2021). Prognostic Cancer Gene Expression Signatures: Current Status and Challenges. Cells.

[13] Colaprico A., Silva T.C., Olsen C., Garofano L., Cava C., Garolini D., Sabedot T.S., Malta T.M., Pagnotta S.M., Castiglioni I. (2016). TCGAbiolinks: An R/Bioconductor Package for Integrative Analysis of TCGA Data. Nucleic Acids Res..

[14] Robinson M.D., McCarthy D.J., Smyth G.K. (2009). EdgeR: A Bioconductor Package for Differential Expression Analysis of Digital Gene Expression Data. Bioinformatics.

[15] Tarazona S., Furió-Tarí P., Turrà D., Di Pietro A., Nueda M.J., Ferrer A., Conesa A. (2015). Data Quality Aware Analysis of Differential Expression in RNA-Seq with NOISeq R/Bioc Package. Nucleic Acids Res..

[16] Ritchie M.E., Phipson B., Wu D., Hu Y., Law C.W., Shi W., Smyth G.K. (2015). Limma Powers Differential Expression Analyses for RNA-Sequencing and Microarray Studies. Nucleic Acids Res..

[17] Belinky F., Nativ N., Stelzer G., Zimmerman S., Stein T.I., Safran M., Lancet D. (2015). PathCards: Multi-Source Consolidation of Human Biological Pathways. Database.

[18] Sun J., Li S., Wang F., Fan C., Wang J. (2019). Identification of Key Pathways and Genes in Pten Mutation Prostate Cancer by Bioinformatics Analysis. BMC Med. Genet..

[19] Milacic M., Beavers D., Conley P., Gong C., Gillespie M., Griss J., Haw R., Jassal B., Matthews L., May B. (2024). The Reactome Pathway Knowledgebase 2024. Nucleic Acids Res..

[20] Ashburber M., Ball C.A., Blake J.A., Botstein D., Butler H., Cherry J.M., Davis A.P., Dolinski K., Dwight S.S., Eppig J.T. (2000). Gene Ontology: Tool for the Unification of Biology. Nat. Genet..

[21] Pico A.R., Kelder T., Van Iersel M.P., Hanspers K., Conklin B.R., Evelo C. (2008). WikiPathways: Pathway Editing for the People. PLoS Biol..

[22] Wishart D.S., Tzur D., Knox C., Eisner R., Guo A.C., Young N., Cheng D., Jewell K., Arndt D., Sawhney S. (2007). HMDB: The Human Metabolome Database. Nucleic Acids Res..

[23] Pedregosa F., Varoquaux G., Gramfort A., Michel V., Thirion B., Grisel O., Blondel M., Prettenhofer P., Weiss R., Dubourg V. (2011). Scikit-Learn: Machine Learning in Python. J. Mach. Learn. Res..

[24] Chen T., Guestrin C. XGBoost: A Scalable Tree Boosting System. Proceedings of the KDD ‘16: The 22nd ACM SIGKDD International Conference on Knowledge Discovery and Data Mining.

[25] Mancini M., Magnani E., Macchi F., Bonapace I.M. (2021). The Multi-Functionality of UHRF1: Epigenome. Nucleic Acids Res..

[26] Kumar V., Vashishta M., Kong L., Wu X., Lu J.J., Guha C., Dwarakanath B.S. (2021). The Role of Notch, Hedgehog, and Wnt Signaling Pathways in the Resistance of Tumors to Anticancer Therapies. Front. Cell Dev. Biol..

[27] National Center for Biotechnology RNF150 Ring Finger Protein 150 [Homo Sapiens (Human)]. https://www.ncbi.nlm.nih.gov/gene?Db=gene&Cmd=DetailsSearch&Term=57484.

[28] Pilleron S., Sarfati D., Janssen-Heijnen M., Vignat J., Ferlay J., Bray F., Soerjomataram I. (2019). Global Cancer Incidence in Older Adults, 2012 and 2035: A Population-Based Study. Int. J. Cancer.

[29] Chung L.M., Ferguson J.P., Zheng W., Qian F., Bruno V., Montgomery R.R., Zhao H. (2013). Differential Expression Analysis for Paired RNA-Seq Data. BMC Bioinform..

[30] Hasugian P.M., Mawengkang H., Sihombing P., Efendi S. Review of High-Dimensional and Complex Data Visualization. Proceedings of the 2023 International Conference of Computer Science and Information Technology (ICOSNIKOM).

[31] Pudjihartono N., Fadason T., Kempa-Liehr A.W., O’Sullivan J.M. (2022). A Review of Feature Selection Methods for Machine Learning-Based Disease Risk Prediction. Front. Bioinform..

[32] Xue J.M., Liu Y., Wan L.H., Zhu Y.X. (2020). Comprehensive Analysis of Differential Gene Expression to Identify Common Gene Signatures in Multiple Cancers. Med. Sci. Monit..

[33] Stevens J.R., Herrick J.S., Wolff R.K., Slattery M.L. (2018). Power in Pairs: Assessing the Statistical Value of Paired Samples in Tests for Differential Expression. BMC Genom..

[34] Hanahan D. (2022). Hallmarks of Cancer: New Dimensions. Cancer Discov..

[35] Yokota J. (2000). Tumor Progression and Metastasis. Carcinogenesis.

[36] Ribatti D., Tamma R., Annese T. (2020). Epithelial-Mesenchymal Transition in Cancer: A Historical Overview. Transl. Oncol..

[37] Fares J., Fares M.Y., Khachfe H.H., Salhab H.A., Fares Y. (2020). Molecular Principles of Metastasis: A Hallmark of Cancer Revisited. Signal Transduct. Target. Ther..

[38] Ma W., Wu H., Chen Y., Xu H., Jiang J., Du B., Wan M., Ma X., Chen X., Lin L. (2024). New Techniques to Identify the Tissue of Origin for Cancer of Unknown Primary in the Era of Precision Medicine: Progress and Challenges. Brief. Bioinform..

