# Supplementary material for: Explainable Machine Learning Models Using Robust Cancer Biomarkers Identification from Paired Differential Gene Expression
Source: Int J Mol Sci. 2024 Nov 19;25(22):12419. doi: 10.3390/ijms252212419 (PMC11594711; doi:10.3390/ijms252212419)
Supplement: Supplementary file 1 [file ijms-25-12419-s001.zip › 2024_11_18_ArticuloCarcinomas_SupplementalFigures.pdf]

Supplementary Figures

Figure S1. Area under the receiver operating characteristic curve (AUC_ROC) and area under the precision-recall curve (AUC_PRC) graphs.

Figure S2. SHAP summary plots, displays SHAP values for each data point in the dataset.

Figure S3. SHAP summary plot, displays SHAP values for each data point in the dataset (breast).

Figure S4. SHAP summary plot, displays SHAP values for each data point in the dataset (colorectal).

Figure S5. SHAP summary plot, displays SHAP values for each data point in the dataset (lung).

Figure S6. SHAP summary plot, displays SHAP values for each data point in the dataset (kidney).

Figure S7. SHAP summary plot, displays SHAP values for each data point in the dataset (gastric).

Figure S8. SHAP summary plot, displays SHAP values for each data point in the dataset (liver).

Figure S9. SHAP summary plot, displays SHAP values for each data point in the dataset (thyroid).

Figure S10. SHAP summary plot, displays SHAP values for each data point in the dataset (uterus).

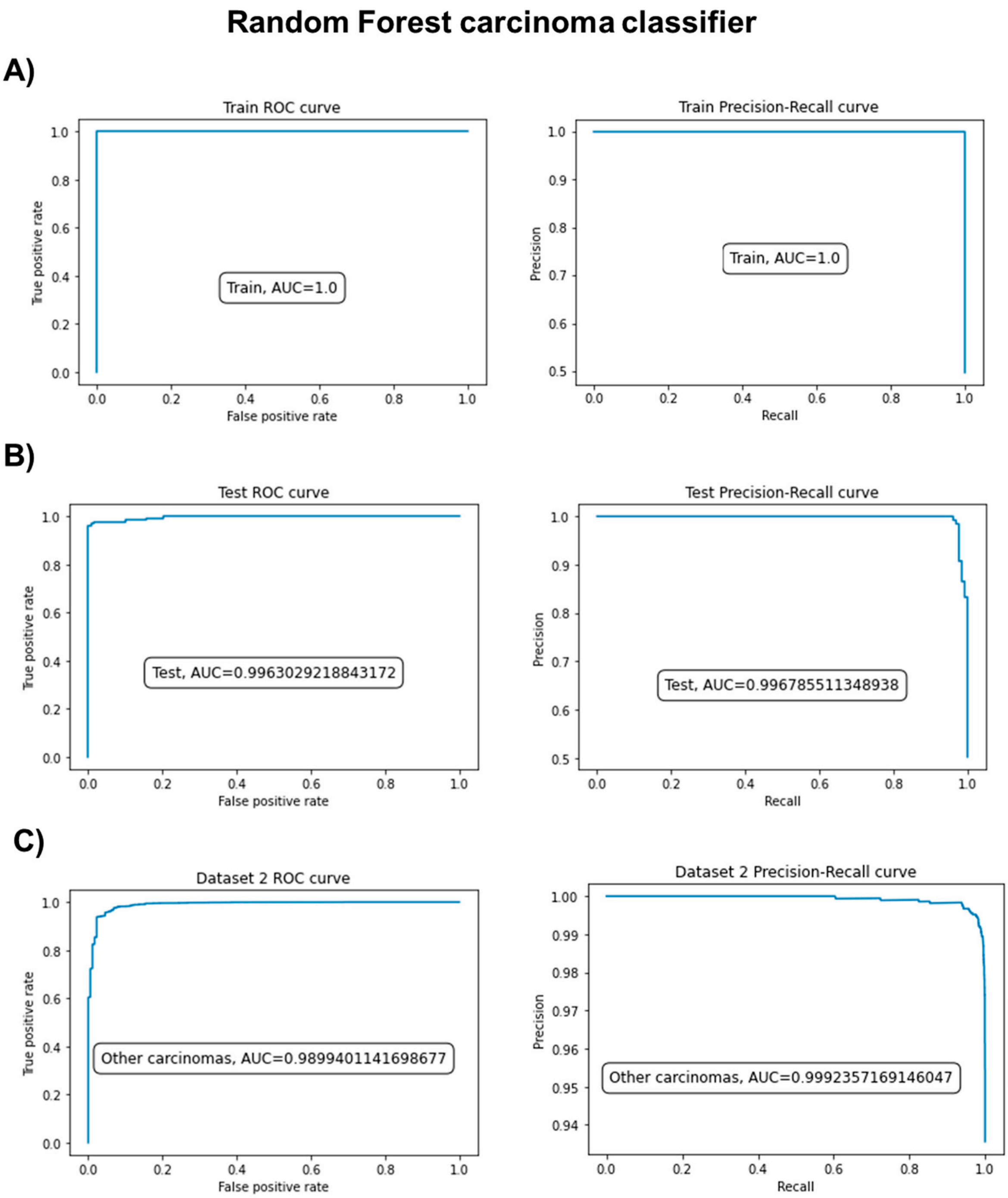

Figure S1. Area under the receiver operating characteristic curve (AUC_ROC) and area under the precision-recall curve (AUC_PRC) graphs. (A) Train set results for AUC_ROC and AUC_PRC. (B) Test set results for AUC_ROC and AUC_PRC. (C) Dataset 2 results for AUC_ROC and AUC_PRC.

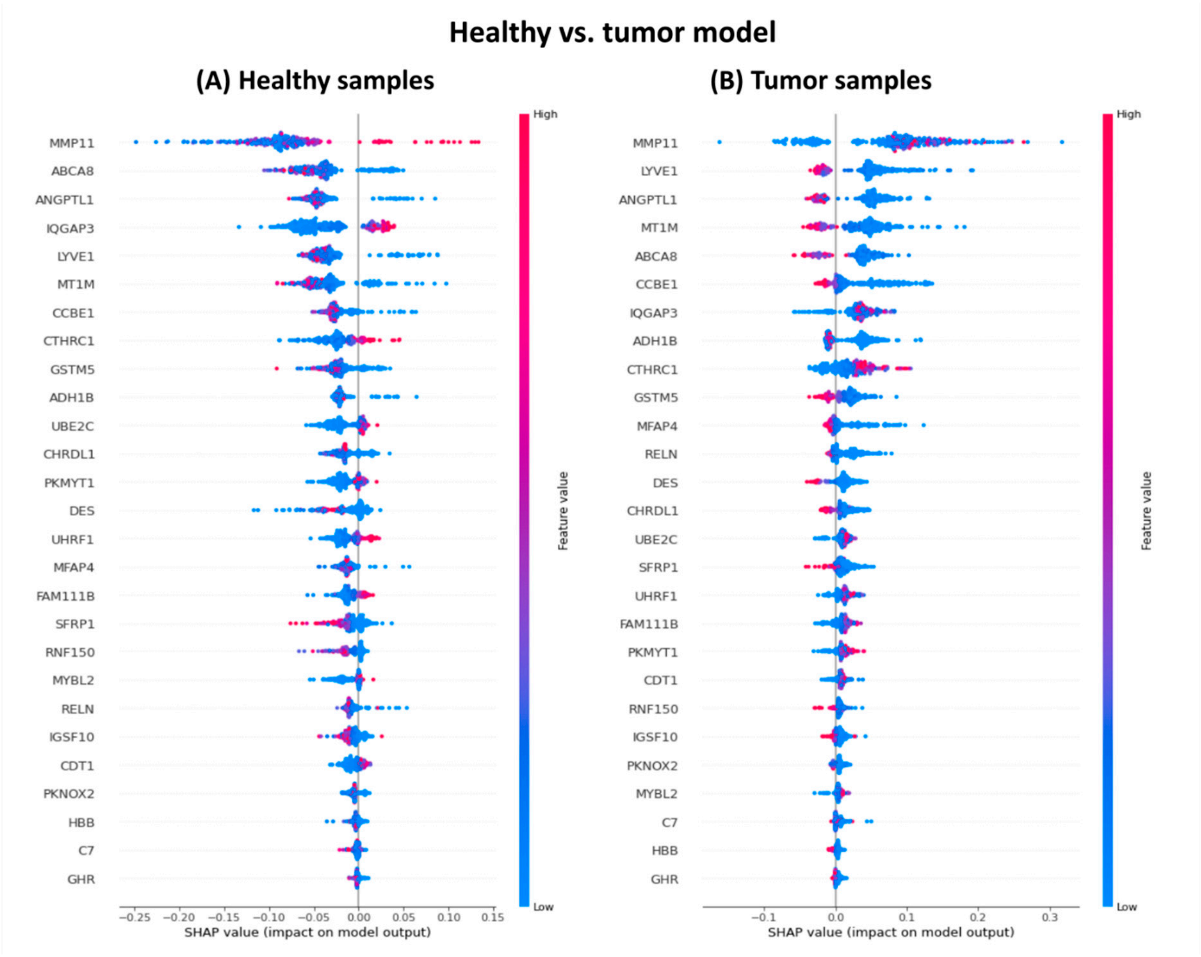

Figure S2. SHAP summary plots, displays SHAP values for each data point in the dataset. Each row corresponds to a feature. Colors indicate feature values —red for high and blue for low. (A) Healthy samples plot, each feature individually categorize each sample (dot), into healthy or tumor category (B) Tumor samples plot, each feature individually categorize each sample (dot), into healthy or tumor category

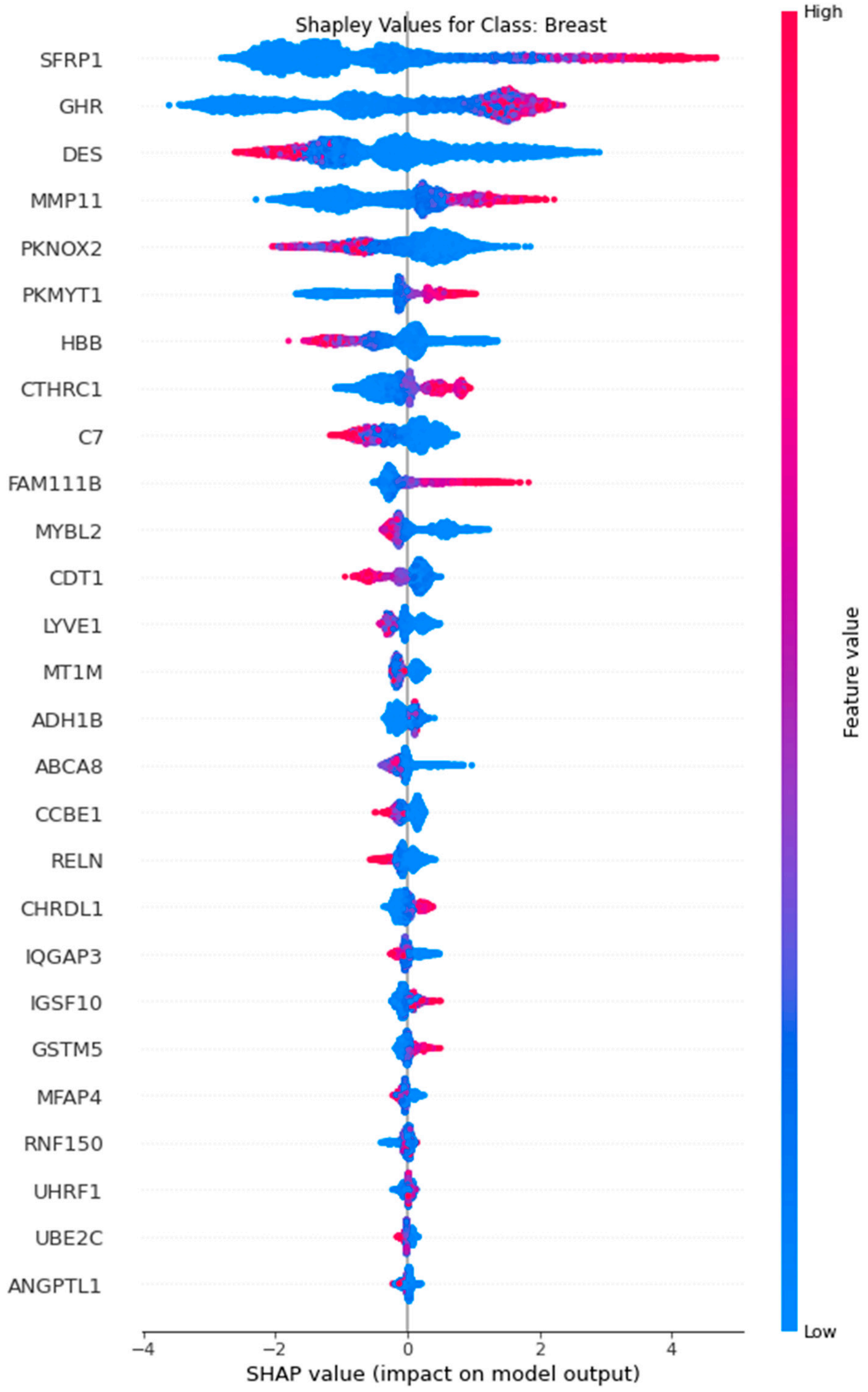

Figure S3. SHAP summary plot, displays SHAP values for each data point in the dataset. Each row corresponds to a feature. Colors indicate feature values —red for high and blue for low. Each feature individually categorize each sample (dot), into breast class (right of the middle line) or non-breast (left of the middle line) category.

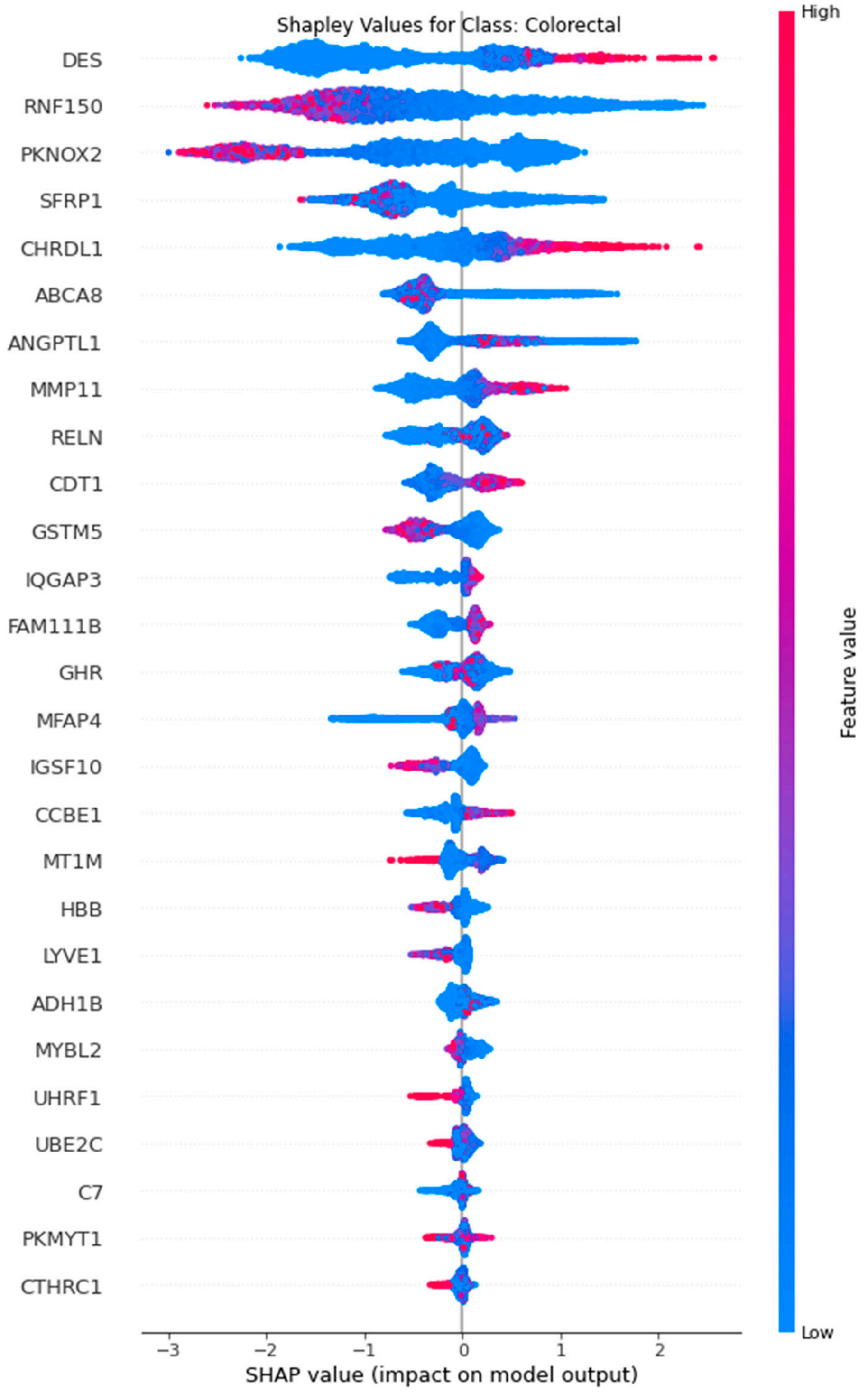

Figure S4. SHAP summary plot, displays SHAP values for each data point in the dataset. Each row corresponds to a feature. Colors indicate feature values —red for high and blue for low. Each feature individually categorizes each sample (dot), into colorectal class (right of the middle line) or non- colorectal (left of the middle line) category.

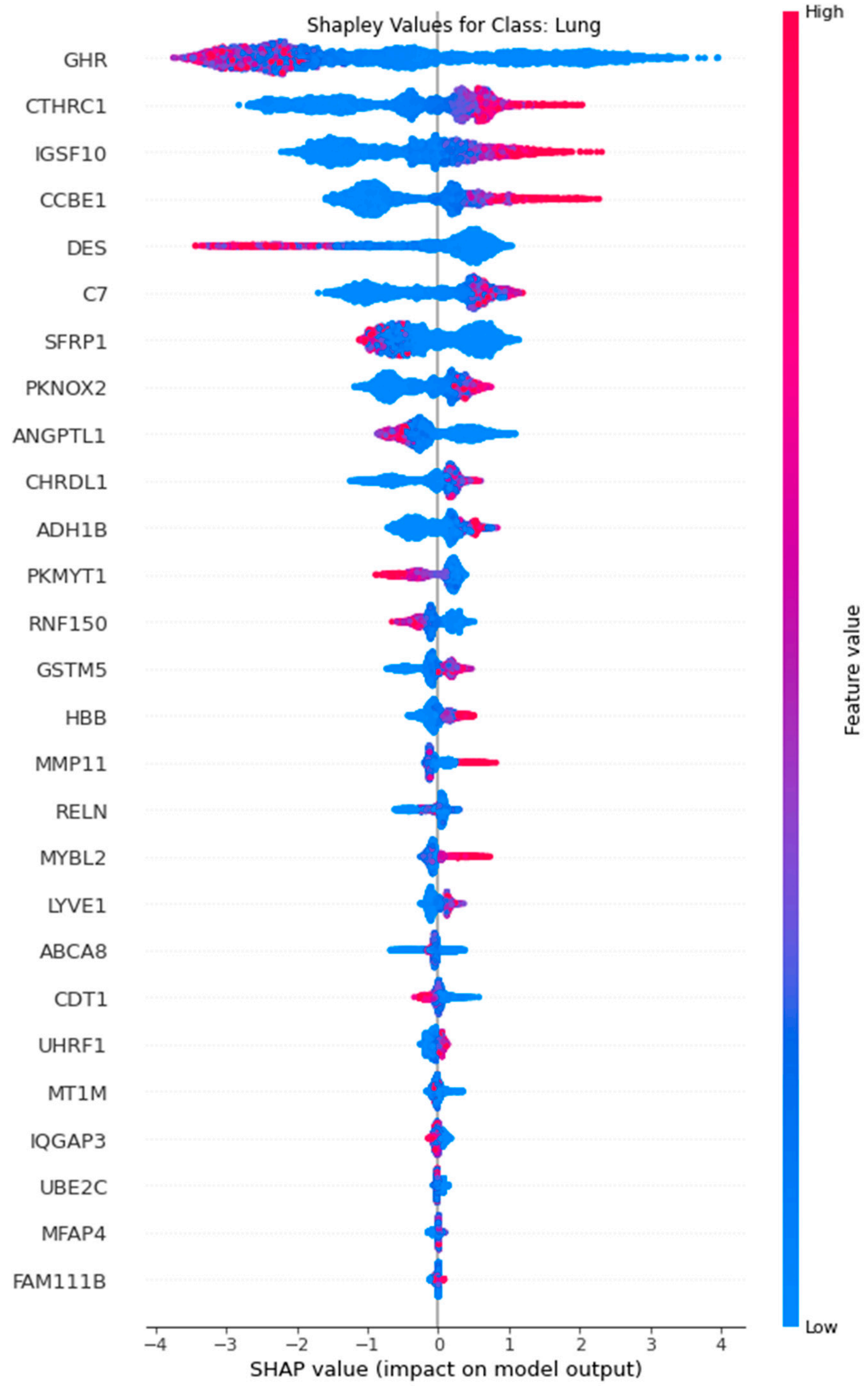

Figure S5. SHAP summary plot, displays SHAP values for each data point in the dataset. Each row corresponds to a feature. Colors indicate feature values —red for high and blue for low. Each feature individually categorizes each sample (dot), into lung class (right of the middle line) or non- lung (left of the middle line) category.

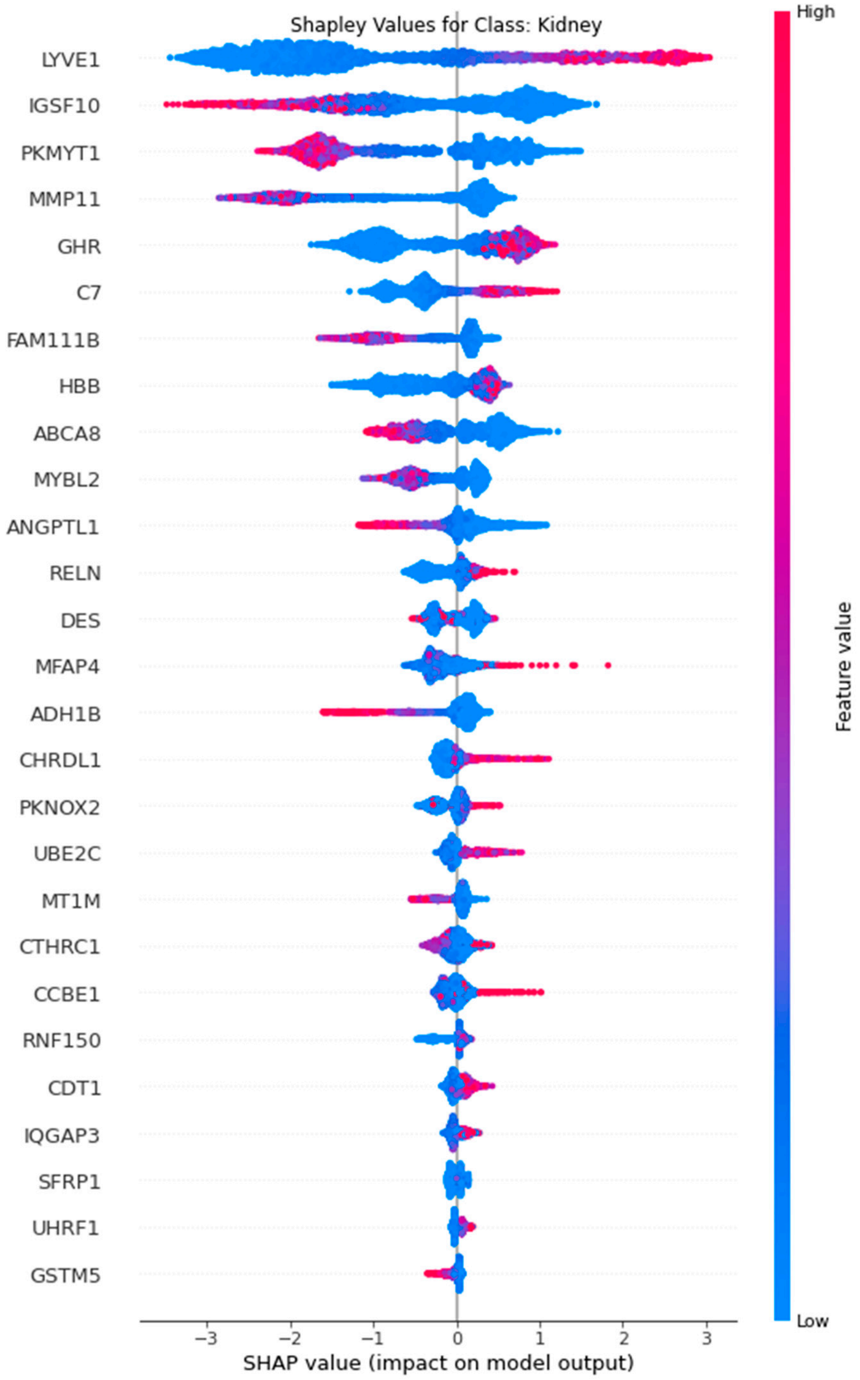

Figure S6. SHAP summary plot, displays SHAP values for each data point in the dataset. Each row corresponds to a feature. Colors indicate feature values —red for high and blue for low. Each feature individually categorizes each sample (dot), into kidney class (right of the middle line) or non- kidney (left of the middle line) category.

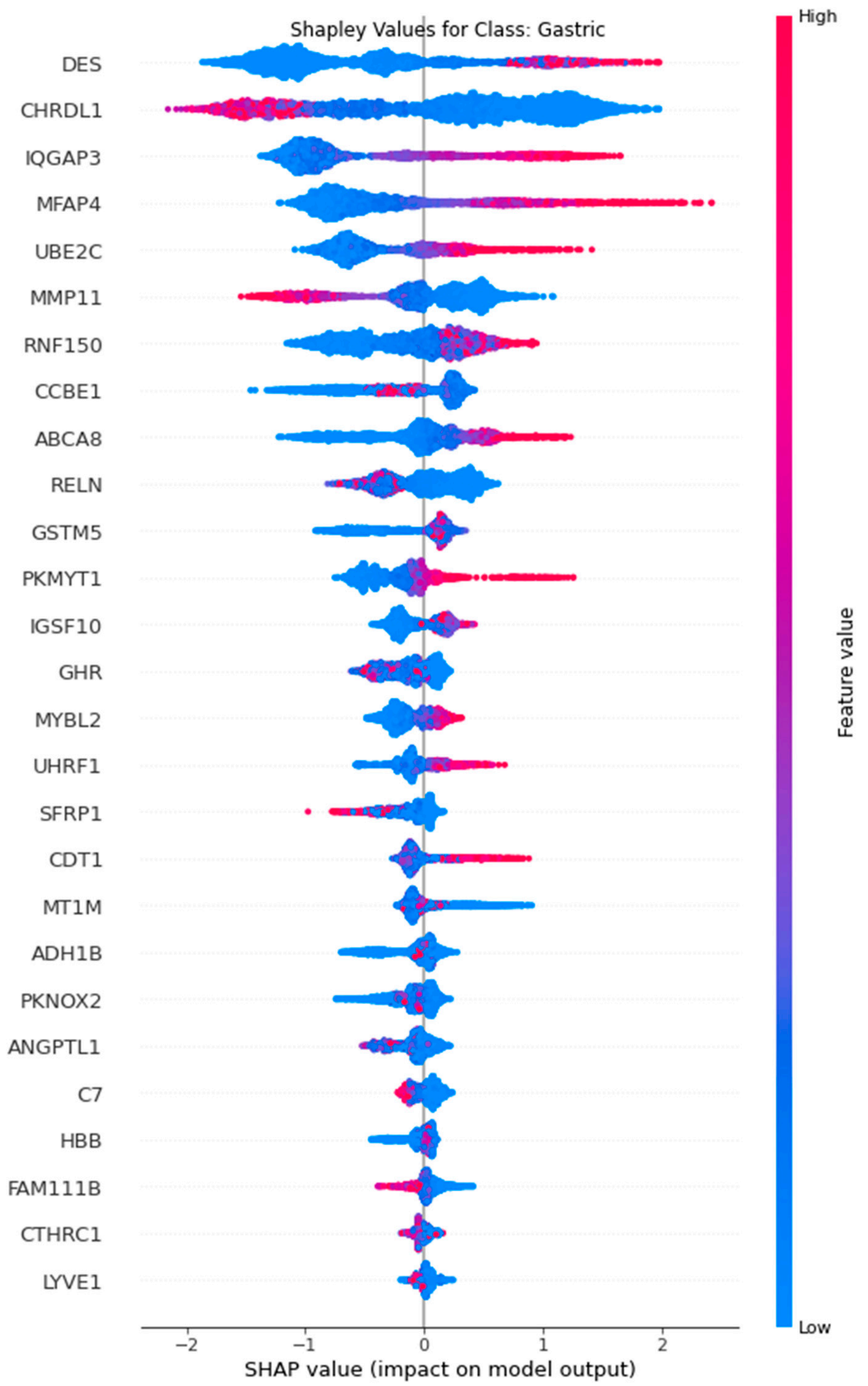

Figure S7. SHAP summary plot, displays SHAP values for each data point in the dataset. Each row corresponds to a feature. Colors indicate feature values —red for high and blue for low. Each feature individually categorizes each sample (dot), into gastric class (right of the middle line) or non- gastric (left of the middle line) category.

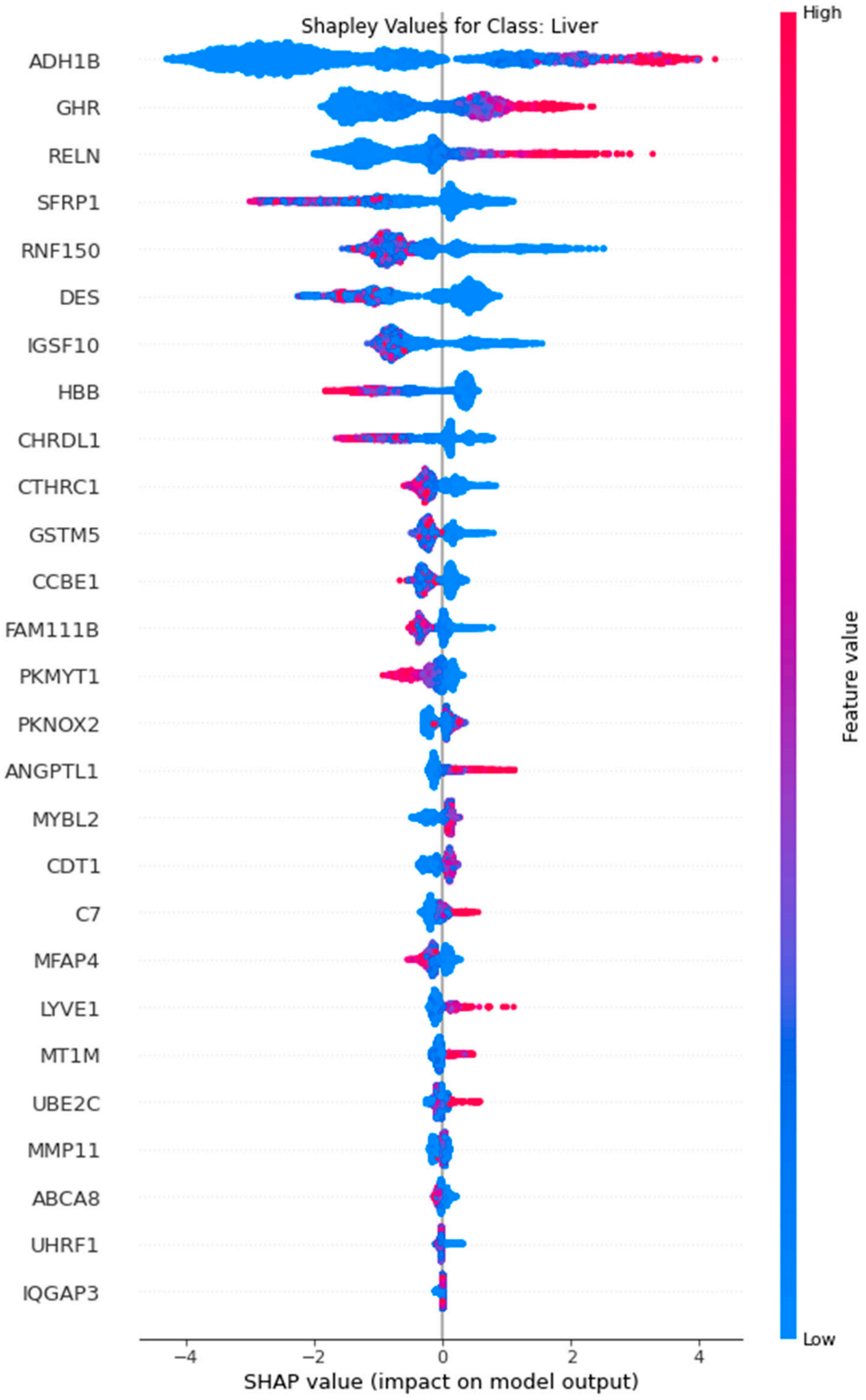

Figure S8. SHAP summary plot, displays SHAP values for each data point in the dataset. Each row corresponds to a feature. Colors indicate feature values —red for high and blue for low. Each feature individually categorizes each sample (dot), into liver class (right of the middle line) or non- liver (left of the middle line) category.

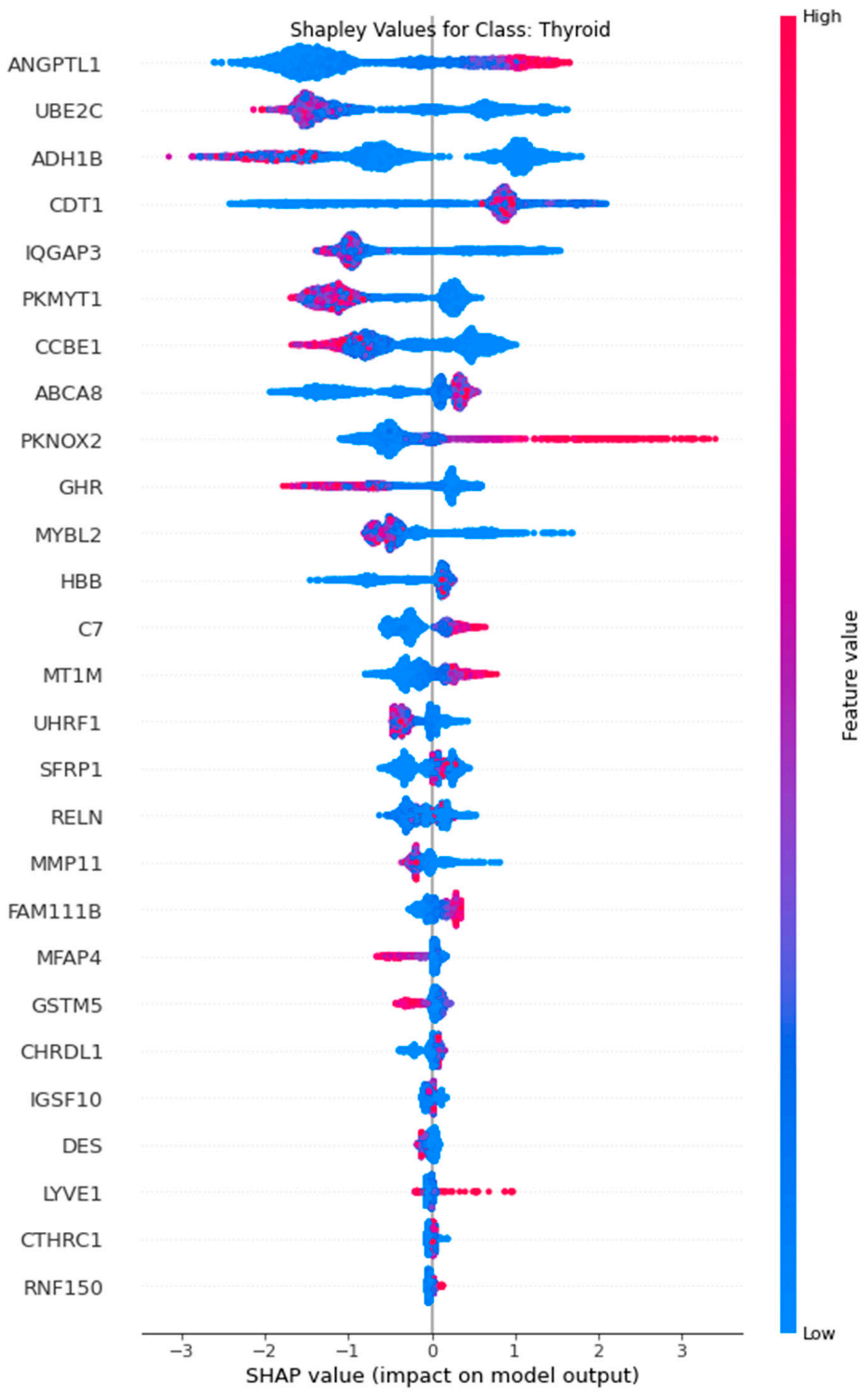

Figure S9. SHAP summary plot, displays SHAP values for each data point in the dataset. Each row corresponds to a feature. Colors indicate feature values —red for high and blue for low. Each feature individually categorizes each sample (dot), into thyroid class (right of the middle line) or non- thyroid (left of the middle line) category.

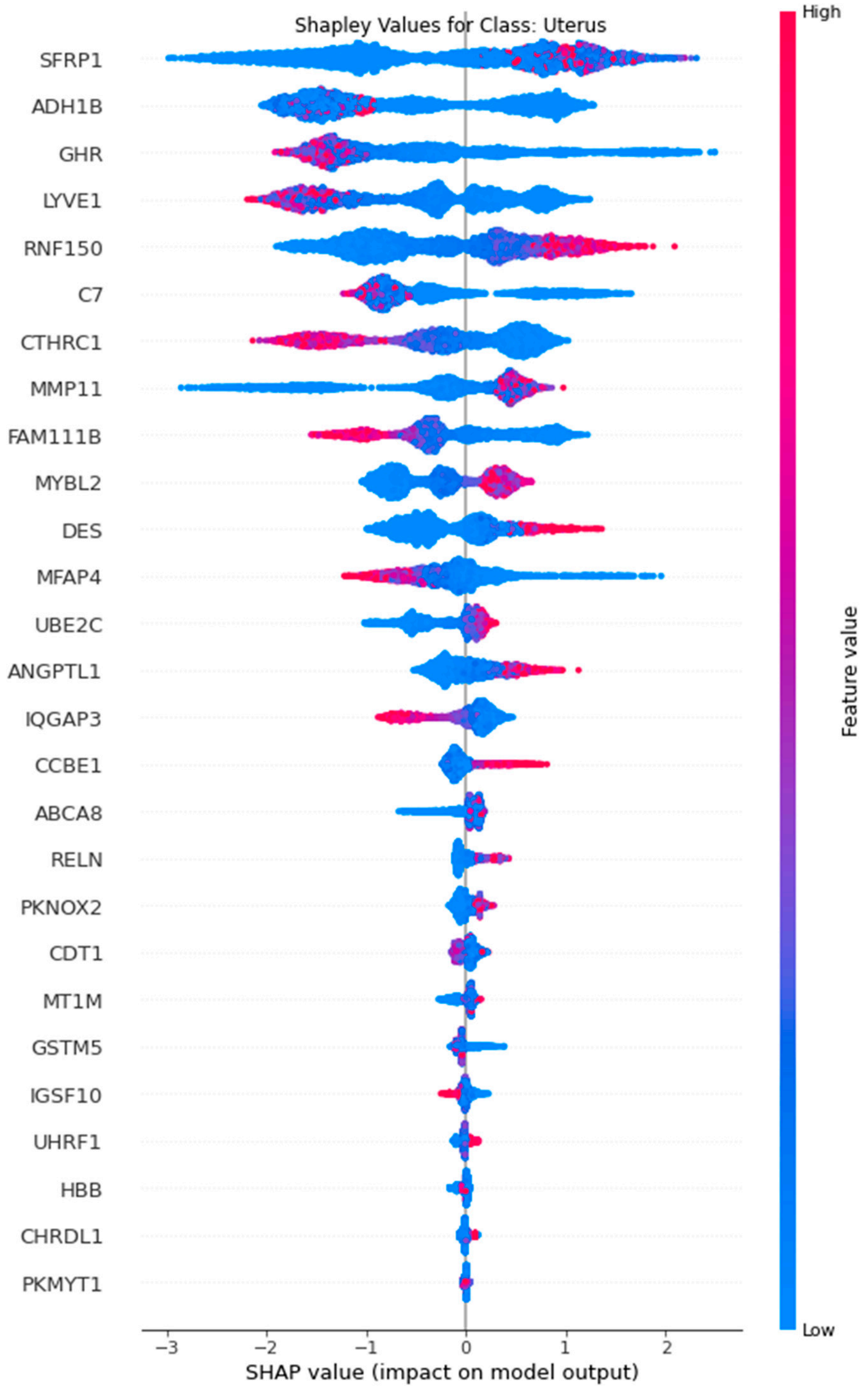

Figure S10. SHAP summary plot, displays SHAP values for each data point in the dataset. Each row corresponds to a feature. Colors indicate feature values —red for high and blue for low. Each feature individually categorizes each sample (dot), into uterus class (right of the middle line) or non- uterus (left of the middle line) category.
