# Supplementary material for: Explainable Machine Learning Models Using Robust Cancer Biomarkers Identification from Paired Differential Gene Expression
Source: Int J Mol Sci. 2024 Nov 19;25(22):12419. doi: 10.3390/ijms252212419 (PMC11594711; doi:10.3390/ijms252212419)
Supplement: Supplementary file 1 [file ijms-25-12419-s001.zip › 2024_11_18_ArticuloCarcinomas_SupplementalMethodology.pdf]

Supplementary methodology

1. The Carcinoma Classifier

2. The Carcinoma tissue-of-origin Classifier

3. Machine Learning Development Model

3.1. The Carcinoma Model

3.2. The Carcinoma tissue-of-origin Model

4. Model Explainability

1. The *Carcinoma Classifier*

Uniform Manifold Approximation and Projection (UMAP) is a method of dimensionality reduction and data clustering. UMAP allows the representation of high-dimensional data in a lower dimensionality while maintaining the structure of the original data. UMAP result (Figure 1) shows clear differences between healthy and tumor specimen sets indicating that they are easily distinguishable.

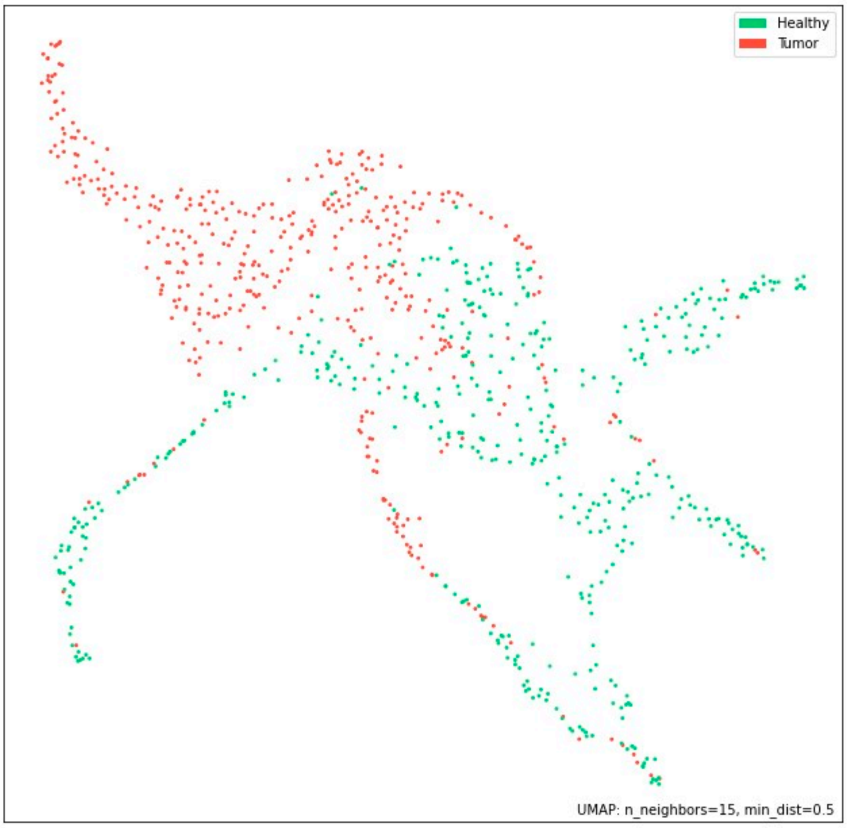

Figure 1. UMAP visualization of healthy and tumor samples. Each point represents a sample, with green points indicating healthy samples and red points indicating tumor samples. The UMAP was generated using n_neighbors=15 and min_dist=0.5.

Initially, a Support Vector Machine (SVM) was employed due to its simplicity and effectiveness in handling classification tasks. While the SVM yielded promising results (Table 1, and Table 2), there was potential for improvement in the model's performance, which is fundamental for cancer detection classifiers.

Table 1. Classification performance metrics for distinguishing tumor and healthy samples. The table shows precision, recall, F1-score, and support for each class. The support column indicates the number of samples in each class: 129 tumor samples and 130 healthy samples.

	Precision	Recall	F1-score	Support
Tumor	0,94	0,91	0,93	129
Healthy	0,92	0,94	0,93	130

Table 2. Classification performance metrics for distinguishing tumor and healthy samples. The table shows accuracy, macro average accuracy, and weighted average accuracy.

Accuracy	0,93
Macro avg	0,93
Weighted avg	0,93

To capture intricate patterns and interactions within the data, in our study, we selected three different ML methods: Random Forest (RF), Multi-Layer Perceptron (MLP), and XGBoost. The specific hyperparameters tested and selected foreach model are listed in supplemental Table S6. In the training set all models achieved outstanding performance, XGBoost had a weighted average precision, recall and f1-score, and accuracy of 99.50%, MLP had a weighted average precision, f1-score and accuracy of 96.03%%, and a recall of 96.04%, RF achieved a perfect score (Table S7, Figure S1).

To assess the feature selection convenience, model's metrics reproducibility and assure the inexistence of over-fitting we repeated the training of the models with three different samplings to create the cross-fold combination using 70% of the sample for training and 30% for test. The results did not change for any of three models (data not shown). Metric's results of XGBoost and RF were similar, and the differences were not significant, although RF gave slightly better results. Given these results, we selected RF as the best model for the general carcinoma classifier with the following parameters (n_estimators: 100, max_depth: None, min_samples_split: 5, min_sample_leaf: 2, bootstrap: False, and default for all the other parameters). In the test set, the metrics for the RF model were also outstanding, it had an accuracy of 98.07%, weighted average precision of 98.10%, recall of 98.07%, and F1-score of 98.07% (Table S8). In addition to metrics above, we evaluated the Area Under the Receiver Operating Characteristic Curve (AUC-ROC) and the Area Under the Precision-Recall Curve (AUC-PRC) to further verify the

model's performance. The RF model achieved an AUC-ROC of 99.63 %, and an AUC-PRC of 99.67%, indicating excellent discrimination ability and precision (Table S8, Figure S1). The excellent metrics obtained on all occasions indicates that the features selected to build the models are robust and relevant, contributing to the strong predictive performance across diverse algorithms.

To assess the carcinoma model's generalization performance, we used two additional external validation datasets (Table S9). Dataset 1 comprised untrained unpaired samples of the same carcinomas as those used for training the classifier. The RF model as expected achieved very high metric values on this dataset, with an accuracy of 99.71%, weighted average recall of 99.71%, and F1-score of 99.85% respectively. We further validated the model using Dataset 2, consisting of paired samples of diverse carcinoma types distinct from those used in the training data. The RF model demonstrated exceptional accuracy (98.28%), precision (98.25%), recall (98.28%), and F1-score (98.26%). The presence of paired data in the Dataset 2, allowed the calculation of the AUC-ROC (98.99%) and the AUC-PRC (99.92%) (Table S9, Figure S1).

2. *The Carcinoma tissue-of-origin Classifier*

To validate the robustness of our approach, we employed the same 27 genes selected by the paired differential expression analysis (DEA) to construct a multiclass model capable of distinguishing the tissue-of-origin for carcinomas. UMAP result shows (Figure 2) clear differences between different carcinomas sets indicating that they are easily distinguishable.

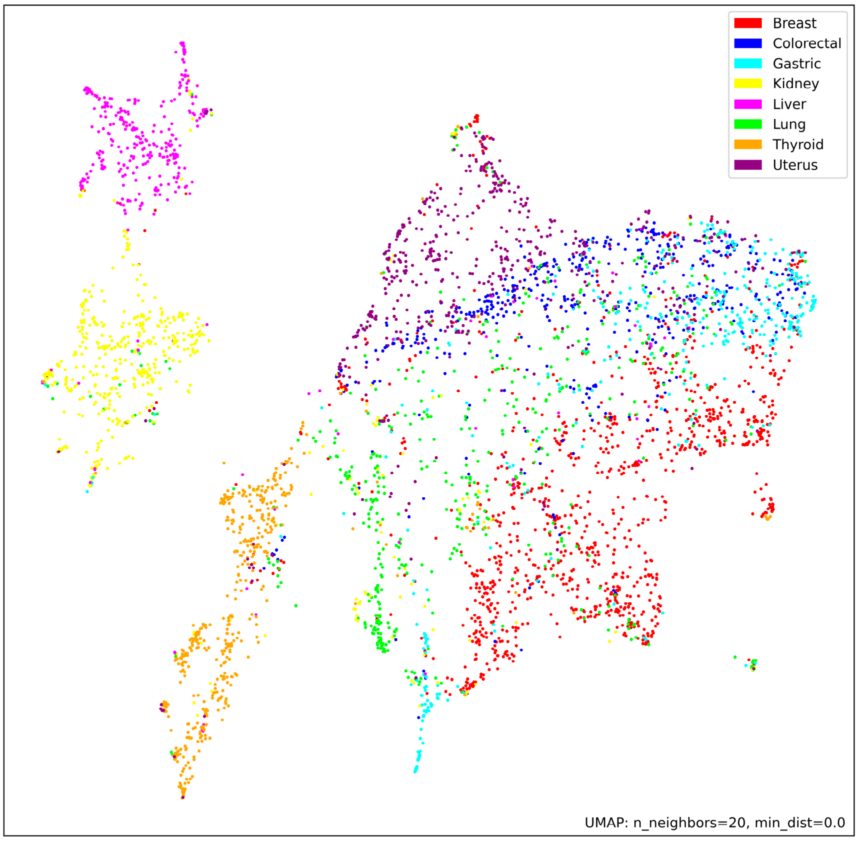

Figure 2. UMAP visualization of carcinoma tissue of origin samples. Each point represents a sample, with color indicating tissue of origin: red for breast, dark blue for colorectal, blue for gastric, yellow for kidney, pink for liver, green for lung, orange for thyroid, and brown for uterus carcinoma. The UMAP was generated using n_neighbors=20 and min_dist=0.0.

Initially, a Support Vector Machine (SVM) was employed due to its simplicity and effectiveness in handling classification tasks. While the SVM yielded promising results (Table 3, and Table 4) there was potential for improvement in the model's performance, which is fundamental for cancer detection classifiers.

Table 3. Classification performance metrics for distinguishing carcinoma tissue of origin. The table shows precision, recall, F1-score, and support for each class. The support column indicates the number of samples in each class: 129 tumor samples and 130 healthy samples.

	Precision	Recall	F1-score	Support
**Breast invasive carcinoma**	0,89	0,91	0,90	111
**Colorectal adenocarcinoma**	0,75	0,88	0,81	48
**Lung adenocarcinoma**	0,84	0,76	0,80	54
**Kidney renal clear cell carcinoma**	0,91	0,93	0,92	54
**Gastric adenocarcinoma**	0,76	0,76	0,76	41
**Liver hepatocellular carcinoma**	0,92	0,89	0,90	37
**Thyroid carcinoma**	1,00	1,00	1,00	51
**Uterine corpus endometrial carcinoma**	0,90	0,80	0,85	56

Table 4. Classification performance metrics for distinguishing carcinoma tissue of origin samples. The table shows accuracy, macro average accuracy, and weighted average accuracy.

Accuracy	0,87
Macro avg	0,87
Weighted avg	0,87

Specifically, as detailed in Section 2.5.2, we exclusively utilized tumor samples to train three algorithms—Random Forest (RF), Multilayer Perceptron (MLP), and XGBoost. The specific hyperparameters tested and selected foreach model are listed in supplemental Table S10. Due to class imbalance, we tried in each algorithm 17 different balancing techniques (Table S11). The borderline Synthetic Minority Over-sampling Technique (SMOTE) was determined to be the most effective strategy

to equilibrate the class imbalance in XGBoost and MLP, while Adasyn was the best technique for Random Forest algorithm (Table S11). Besides, there were 36 other combinations of algorithm-balancing techniques with an average f1-score of >90%, 18 of them above >95% (Table S11).

Given these results, we selected borderline SMOTE XGBoost as the best model with the following hyperparameters (learning_rate: 0.3, n_estimators: 100, max_depth: 3, min_child_weight: 1, subsample: 0.7, colsample_bytree= 0.7, gamma= None, and default for all the other parameters). Model's performance over the train set achieved an average precision, recall, and f1 score of 99.97%, specificity of 99.99%, average GEO of 99.98%, and an average IBA of 99.96% (Table S11). GEO and IBA assess not just the overall accuracy but the fairness and effectiveness of the model across different classes, especially in imbalanced problems.

In the test set, the metrics for the model were also outstanding, we used 452 samples that were left aside from the beginning as test set. Average precision was 95.04%, average recall was 94.91%, average F1 was 94.92%, average GEO was 96.98%, and average IBA was 93.68% (Table S12). The excellent metrics obtained on all occasions indicates that the features selected to build the models are robust and relevant, contributing to the strong predictive performance across diverse algorithms.

3. Machine Learning Development Model

3.1. The Carcinoma Model

For the carcinoma classifier we used the genes selected in section 2.3. The gene expression values were obtained from the samples specified in section 2.1.1 being allocated into two independent sets for distinct phases of the model development: 605 samples were used for training purposes, and 259 samples were utilized for independent testing. The paired nature of these samples ensured a balanced representation of both tumor and healthy tissues across all eight carcinoma types in the training and testing datasets.

Initially, a Support Vector Machine (SVM) was employed due to its simplicity and effectiveness in handling classification tasks. While the SVM yielded promising results, there was potential for improvement in the model's performance. To explore this, more complex algorithms were subsequently implemented, including Random Forest (RF), XGBoost, and Multi-Layer Perceptron (MLP). These advanced methods were chosen for their ability to capture intricate patterns and interactions within the data, ultimately aiming to enhance the predictive accuracy and robustness of the model.

In each algorithm, after establishing the optimal hyperparameters, model fitting was executed, and the best model was selected based on its performance metrics. Model evaluation included standard measures such as precision, recall, F1 score,

area under the receiver operating characteristic curve (AUC_ROC), and area under the precision-recall curve (AUC_PRC)[23,25,26].

3.2. The Carcinoma tissue-of-origin Model

For the carcinoma tissue of origin classifier, we used the genes selected in section 2.3 and the samples indicated in section 2.1.2. A total of 4,513 samples (Table 1) were allocated into three balanced, independent sets for distinct phases of the model development: hyperparameter optimization (n=407), model fitting (n=3,654), and test (n=452). To address the dataset's imbalance, various sampling techniques were tested, including oversampling, undersampling, and combined methods.

Initially, a Support Vector Machine (SVM) was employed due to its simplicity and effectiveness in handling classification tasks. While the SVM yielded promising results, there was potential for improvement in the model's performance. To explore this, more complex algorithms were subsequently implemented, including Random Forest (RF), XGBoost, and Multi-Layer Perceptron (MLP). These advanced methods were chosen for their ability to capture intricate patterns and interactions within the data, ultimately aiming to enhance the predictive accuracy and robustness of the model.

In each algorithm, after establishing the optimal hyperparameters, model fitting was executed to set the parameters, selecting the best model based on its performance metrics. Model evaluation included standard measures such as precision, recall, F1 score, area under the receiver operating characteristic curve (AUC-ROC), and area under the precision-recall curve (AUC-PRC). Additionally, metrics tailored for multiclass settings, such as the Geometric Mean Score (GEO)[27] and the Index of Balanced Accuracy (IBA)[27,28], were employed.

4. Explainability

In the context of healthcare, models not only need to perform well but also provide insights into the biological and logical foundations of their decisions. One method that has gained prominence is SHAP (Shapley Additive exPlanations)[12]. SHAP values quantify the impact of individual features on a model's output, enabling both global and local interpretations of feature importance. Global interpretations involve aggregating SHAP values across all samples, shedding light on the overall significance of each gene within the model[29]. Conversely, local interpretations focus on how specific genes influence predictions for individual samples. To enhance model explainability, we generated both global and local summary plots for the carcinoma classifier. These plots prioritize genes based on their importance in distinguishing between carcinoma and healthy tissue. Additionally, for the

carcinoma tissue of origin classifier, we created local summary plots for each of the eight carcinoma types, highlighting the key genes crucial for characterizing specific carcinoma tissue types.
